# Serine protease Rv2569c facilitates transmission of *Mycobacterium tuberculosis* via disrupting the epithelial barrier by cleaving E-cadherin

**DOI:** 10.1371/journal.ppat.1012214

**Published:** 2024-05-09

**Authors:** Xinxin Zang, Jiajun Zhang, Yanyan Jiang, Tingting Feng, Yingying Cui, Hui Wang, Ziyin Cui, Guanghui Dang, Siguo Liu

**Affiliations:** State Key Laboratory for Animal Disease Control and Prevention, Division of Bacterial Diseases, Harbin Veterinary Research Institute, Chinese Academy of Agricultural Sciences, Harbin, PR China; Portland VA Medical Center, Oregon Health and Science University, UNITED STATES

## Abstract

Epithelial cells function as the primary line of defense against invading pathogens. However, bacterial pathogens possess the ability to compromise this barrier and facilitate the transmigration of bacteria. Nonetheless, the specific molecular mechanism employed by *Mycobacterium tuberculosis* (*M*.*tb*) in this process is not fully understood. Here, we investigated the role of Rv2569c in *M*.*tb* translocation by assessing its ability to cleave E-cadherin, a crucial component of cell-cell adhesion junctions that are disrupted during bacterial invasion. By utilizing recombinant Rv2569c expressed in *Escherichia coli* and subsequently purified through affinity chromatography, we demonstrated that Rv2569c exhibited cell wall–associated serine protease activity. Furthermore, Rv2569c was capable of degrading a range of protein substrates, including casein, fibrinogen, fibronectin, and E-cadherin. We also determined that the optimal conditions for the protease activity of Rv2569c occurred at a temperature of 37°C and a pH of 9.0, in the presence of MgCl_2_. To investigate the function of Rv2569c in *M*.*tb*, a deletion mutant of Rv2569c and its complemented strains were generated and used to infect A549 cells and mice. The results of the A549-cell infection experiments revealed that Rv2569c had the ability to cleave E-cadherin and facilitate the transmigration of *M*.*tb* through polarized A549 epithelial cell layers. Furthermore, *in vivo* infection assays demonstrated that Rv2569c could disrupt E-cadherin, enhance the colonization of *M*.*tb*, and induce pathological damage in the lungs of C57BL/6 mice. Collectively, these results strongly suggest that *M*.*tb* employs the serine protease Rv2569c to disrupt epithelial defenses and facilitate its systemic dissemination by crossing the epithelial barrier.

## Author summary

*Mycobacterium tuberculosis* (*M*.*tb*) *causes* tuberculosis (TB), a highly contagious respiratory disease and a leading cause of death worldwide. Following the inhalation of aerosol particles, the bacteria often establish a persistent infection within the lungs, leading to the development of the characteristic pulmonary TB syndrome. Additionally, *M*.*tb* can breach alveolar barrier, thereby initiating extrapulmonary TB. The pathogenesis of *M*.*tb* heavily relies on systemic dissemination, although the precise underlying mechanism remains incompletely elucidated. The alveolar epithelial barrier, composed of tight junctions and adherens junctions, plays a crucial role in safeguarding against invading pathogens. In this study, we present groundbreaking discoveries pertaining to the extracellular serine protease Rv2569c of *M*.*tb*, which facilitates bacterial transmigration through the cleavage of E-cadherin. Furthermore, we ascertain that Rv2569c elicits profound pathological harm and enhances *M*.*tb* colonization within murine pulmonary tissues. These findings contribute to a deeper comprehension of the pathogenic mechanisms employed by *M*.*tb* and hold potential for informing future strategies aimed at controlling TB infections.

## Introduction

Tuberculosis (TB) is a severe contagious infectious disease caused by *Mycobacterium tuberculosis* (*M*.*tb*) [1]. Prior to the onset of the coronavirus (COVID-19) pandemic, TB was the primary cause of morbidity and mortality resulting from a single infectious agent, surpassing HIV/AIDS [2,3]. Despite the administration of the attenuated vaccine, Bacillus Calmette–Guérin (BCG) vaccine, approximately one-third of the global population has contracted TB [4,5]. *M*.*tb* is a respiratory pathogen that primarily spreads via aerosolized droplet nuclei from person to person [6,7]. Upon inhalation of aerosol particles, bacteria often establish a stable infection in the lungs, which results in the development of the classic syndrome of pulmonary TB and transmission across the alveolar barrier to trigger systemic dissemination [8,9]. Extrapulmonary TB can develop in all other tissues and organs, such as the spine, kidneys, brain, and lymph nodes [10–12].

Systemic dissemination is a crucial aspect of the pathogenesis of *M*.*tb* [13,14]. To access other tissues, *M*.*tb* must first cross the alveolar epithelial barrier [15,16]. The alveolar epithelial barrier is essential in defending against invading pathogens and toxins by performing a biological barrier function through the presence of mucociliary clearance and intercellular junctions [16,17]. Tight junctions (TJs) and adherens junctions (AJs) are intercellular junctions that contribute to the formation and maintenance of this barrier [18,19]. TJs consist of several protein elements, such as tricellulin, occludin, claudin, and junctional adhesion molecule, which are transmembrane proteins linked to the actin cytoskeleton between neighboring cells [20–22]. AJs play a crucial role in regulating various cellular processes, including cell adhesion, cell polarization, intracellular signaling, and transcriptional regulation [19,23]. E-cadherin, a Ca^2+^-dependent transmembrane glycoprotein, comprises an extracellular domain, a transmembrane domain, and a short intracellular domain [24]. As the primary constituent of AJs, E-cadherin is essential for maintaining epithelial barrier integrity and regulating numerous fundamental aspects of epithelial biology [25].

Numerous pathogenic bacteria, such as *Campylobacter jejuni*, *Actinobacillus pleuropneumoniae*, *Helicobacter pylori*, *Yersinia enterocolitica*, and *Candida albicans*, have devised tactics to overcome the epithelial barrier and establish short- or long-term infections in deeper tissues [26–29]. These takeover strategies involve the breakdown of E-cadherin by extracellular proteases produced by the pathogen, enabling the bacteria to traverse the epithelial barrier and disseminate systemically.

The role of extracellular proteases in the destruction of intercellular junction proteins and crossing of the epithelial barrier is deemed crucial. Nonetheless, the pathogenic mechanism employed by *M*.*tb* to cleave E-cadherin and traverse the alveolar epithelial barrier remains unknown. This study presented evidence that the serine protease Rv2569c cleaves E-cadherin, thereby promoting bacterial transmigration and causing severe pathological damage to the lungs of mice.

## Results

### Identification and location of Rv2569c

**Fig 1:**
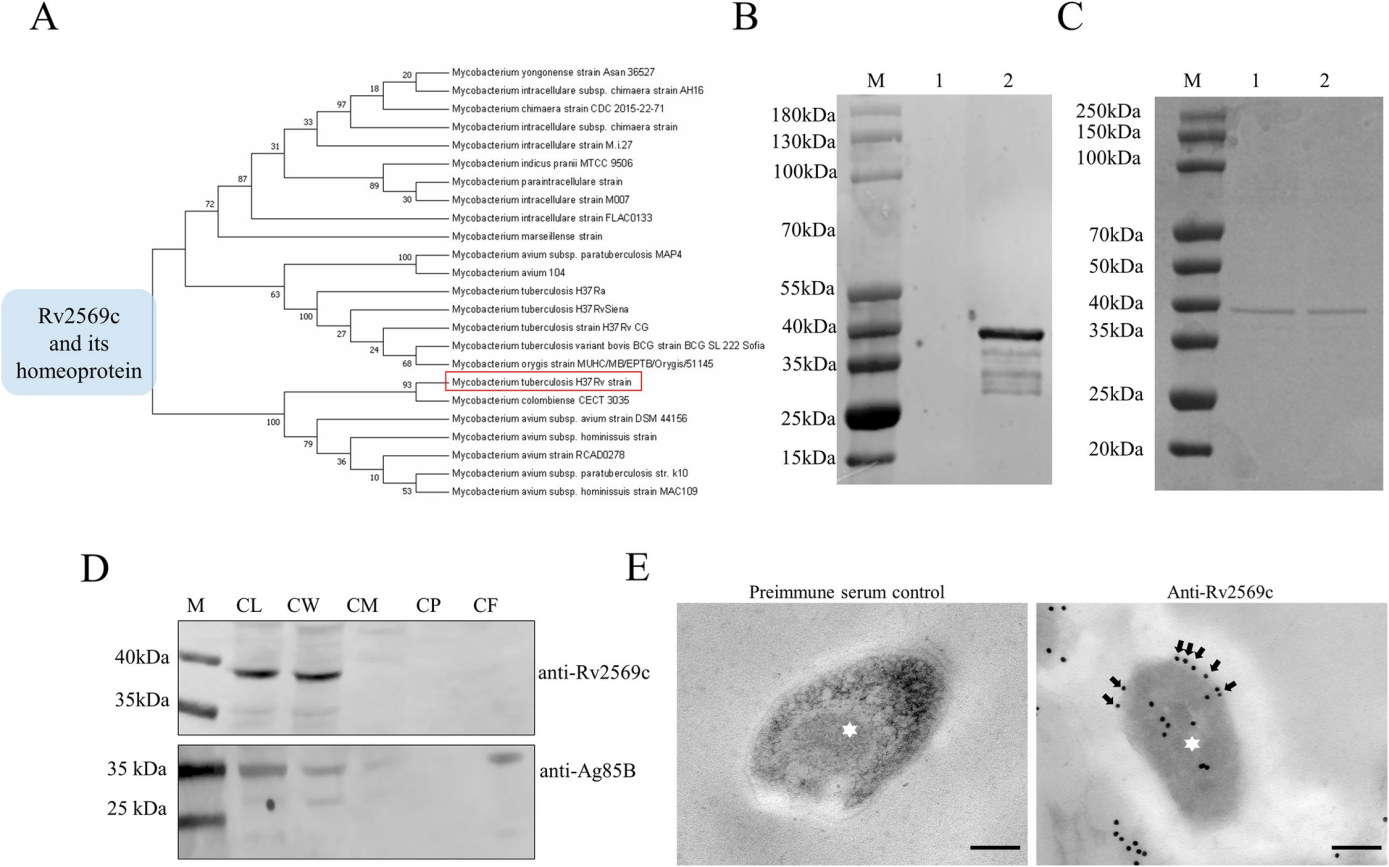
Purification, identification, and subcellular localization of the Rv2569c protein. (A) Phylogenetic tree analysis of the Rv2569c protein using MEGA 7 software. (B) Identification of the Rv2569c protein by western blot. Lane M: protein marker; Lane 1: induced empty vector pET-28a; Lane 2: induced pET28a-Rv2569c. (C) Ni-column affinity chromatography purification of the Rv2569c protein by SDS-PAGE. Lane M: protein marker; Lanes 1 and 2: purified Rv2569c protein. (D) Subcellular localization of the Rv2569c protein in H37Rv. CL: whole bacterial component; CW: cell wall; CM: cell membrane; CP: cytoplasm; CF: culture supernatant. (E) Immunoelectron microscopy analysis of Rv2569c of H37Rv cells on ultrathin sections. Pre-immune serum served as negative control for the anti-Rv2569c antibody. Asterisks represent the H37Rv cells. Arrows represent the Rv2569c protein. Scale bars, 200 nm.

Through the utilization of conserved domain analysis, we determined that Rv2569c—a member of the YebA superfamily—exhibited characteristics of a potential protease [30]. Further investigation via multiple sequence alignment of mycobacteria genomes revealed that a homolog of Rv2569c was a prevalent protein within mycobacteria (Fig 1A). All associated data was exhibited in the GitHub repository (https://github.com/zangxinx/Rv2569c-phylogenetic-tree). To evaluate the functionality of Rv2569c, the recombinant plasmid pET28a-Rv2569c was introduced into *Escherichia coli* (*E*. *coli*) BL21 and subsequently confirmed through western blot analysis, which showed a protein band with a molecular weight of 38 kDa (Fig 1B). Subsequently, Rv2569c protein was purified by Ni-column affinity chromatography, and Rv2569c protein with more than 98% purity was obtained from the supernatants of bacterial cultures after ultrasonication (Fig 1C). To ascertain the subcellular localization of Rv2569c in H37Rv, we generated an anti-Rv2569c polyclonal antibody and employed western blot analysis to examine the subcellular fractions. Ag85B, which served as a marker protein, was found in the cell wall, cell membrane, and secreted protein. Our findings indicated that Rv2569c was predominantly localized on the cell wall (Fig 1D). Additionally, the immunoelectron microscopy results confirmed the presence of Rv2569c on the surface of H37Rv cells (Fig 1E). Collectively, these results demonstrate that Rv2569c is an extracellular protein in H37Rv.

### Rv2569c has a serine protease activity

**Fig 2:**
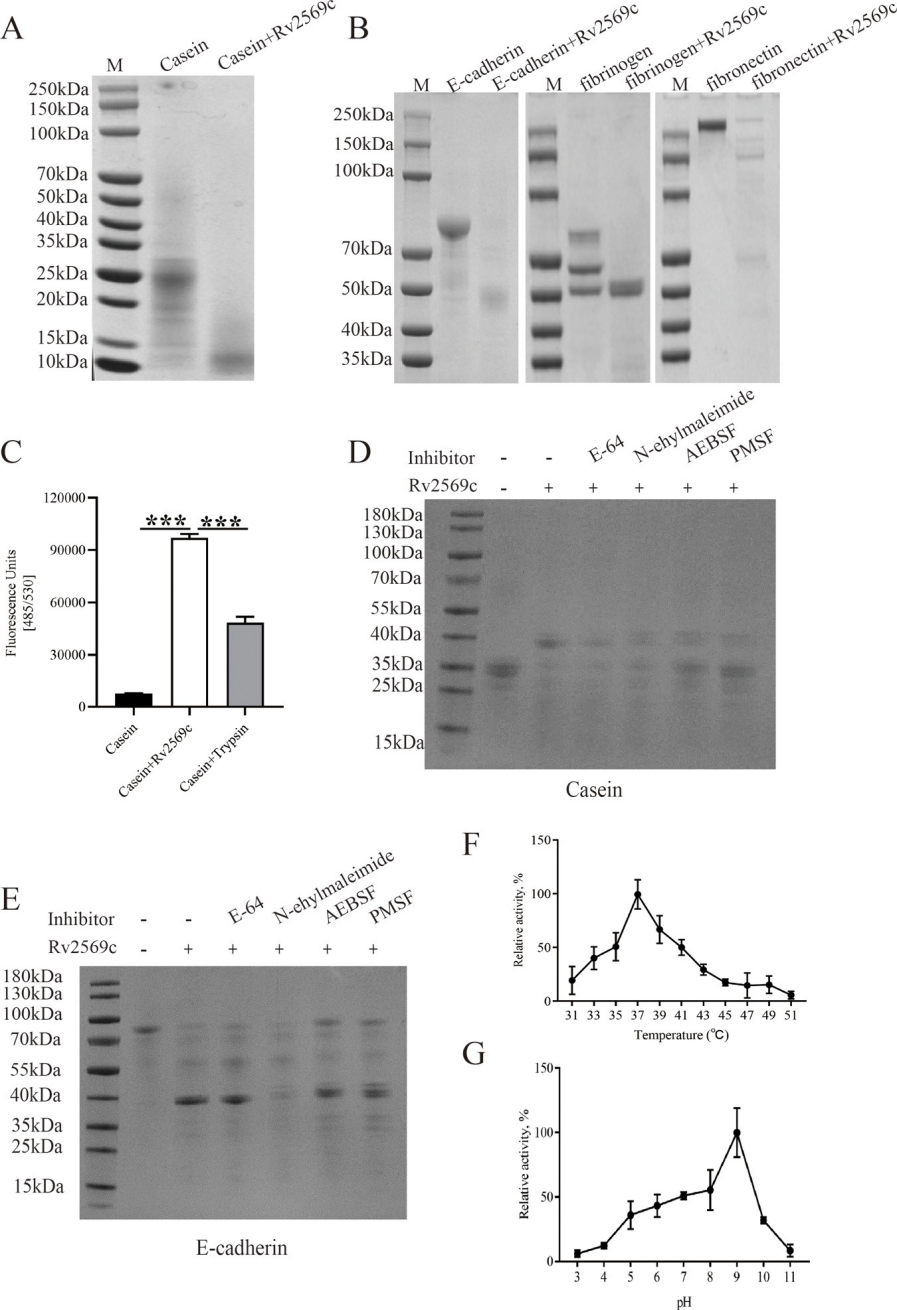
Characterization of the Rv2569c protease activity. (A) The protease activity of Rv2569c was evaluated by digesting casein as the substrate. (B) Various protein substrates (E-cadherin, fibrinogen, and fibronectin) were degraded by purified Rv2569c protein. (C) Quantitative analysis of FITC-labeled casein digestion by Rv2569c protein. Trypsin was considered as a positive control. (D) and (E) The category of Rv2569c protease was determined by incubating casein or E-cadherin with different chemical inhibitors. (F) The effect of temperature on the activity of Rv2569c protease was analyzed quantitatively by spectrophotometry. (G) The effect of pH on the activity of Rv2569c protease was analyzed quantitatively by spectrophotometry. The error bars represent the SEM of three independent experiments. One-way ANOVA, followed by Bonferroni**’**s multiple-comparison post-hoc test (***P < 0.001). All assays were repeated three times.

To determine the protease activity of Rv2569c, casein, fibrinogen, fibronectin, and E-cadherin were employed as substrates. We found that Rv2569c protein exhibited the ability to degrade casein, fibrinogen, fibronectin, and E-cadherin (Fig 2A, 2B and 2C). The proteolytic activity of Rv2569c in degrading E-cadherin and casein was entirely inhibited by AEBSF (a serine protease inhibitor) and PMSF (a serine and cysteine protease inhibitor) (Fig 2D and 2E). The proteolytic activity of Rv2569c was not affected by the presence of E-64 and N-ethylmaleimide, both of which are cysteine protease inhibitors. These findings suggest that Rv2569c has a serine protease activity and is capable of degrading various protein substrates.

### Effect of temperature, pH, and divalent cations on Rv2569c protease activity

To determine the optimal reaction conditions for Rv2569c protease, experiments were conducted across a broad temperature range (31°C–51°C). We found that the optimal temperature for Rv2569c protease was 37°C (Fig 2F). In the pH range of 3.0–9.0, the maximum protease activity of Rv2569c was found at pH 9.0 (Fig 2G). Furthermore, the effect of various divalent cation salts on the protease activity of Rv2569c was evaluated (as shown in S2 Table). The protease activity was significantly improved in the presence of 5 mM MgCl_2_, while other metal ion salts, namely MnCl_2_, BaCl_2_, and CaCl_2_, caused a slight enhancement in the protease activity of Rv2569c. In contrast, NiCl_2_ showed a slight inhibition of Rv2569c protease activity. These results indicate that the optimal conditions for enzymatic activity of Rv2569c are a temperature of 37°C and a pH of 9.0, with the presence of MgCl_2_.

### Construction of Rv2569c-deletion mutant, complementation in H37Rv and H37Ra strains, and recombinant Rv2569c_Ms strains

To further determine the role of Rv2569c in *M*.*tb* with regards to cleaving E-cadherin, Rv2569c-deficient strains (H37RvΔRv2569c and H37RaΔRv2569c) were generated using the homologous recombination method (S1A Fig). They were confirmed through PCR identification and southern blot analysis, which showed that the *rv2569c* gene had been substituted by a sacB-hyg cassette in H37Rv (S1B and S1C Fig) and H37Ra (S1D and S1E Fig). Complementation assays were also conducted, and PCR analysis confirmed the presence of the introduced Rv2569c construct in the H37RaΔRv2569c + Rv2569c and H37RvΔRv2569c + Rv2569c strains (S2A Fig). Subsequently, the recombinant plasmid pAIN-Rv2569c was transformed into *Mycolicibacterium smegmatis* (Ms) to generate the overexpression strain Rv2569c_Ms. The expression of the Rv2569c protein was evaluated via western blot analysis (S2B Fig). The H37RvΔRv2569c showed no significant differences in colony morphology and the growth rate relative to the H37Rv and H37RvΔRv2569c + Rv2569c strains (S3A and S3B Fig). Similar observations were made in H37Ra, H37RaΔRv2569c, and H37RaΔRv2569c + Rv2569c strains (S3C and S3D Fig), and pAIN_Ms and Rv2569c_Ms strains (S3E and S3F Fig). Taken together, these results suggest that the deletion of Rv2569c does not alter bacterial growth and colony morphology under standard culture conditions.

### Rv2569c facilitates *M*.*tb* to cleave E-cadherin *in vitro*

**Fig 3:**
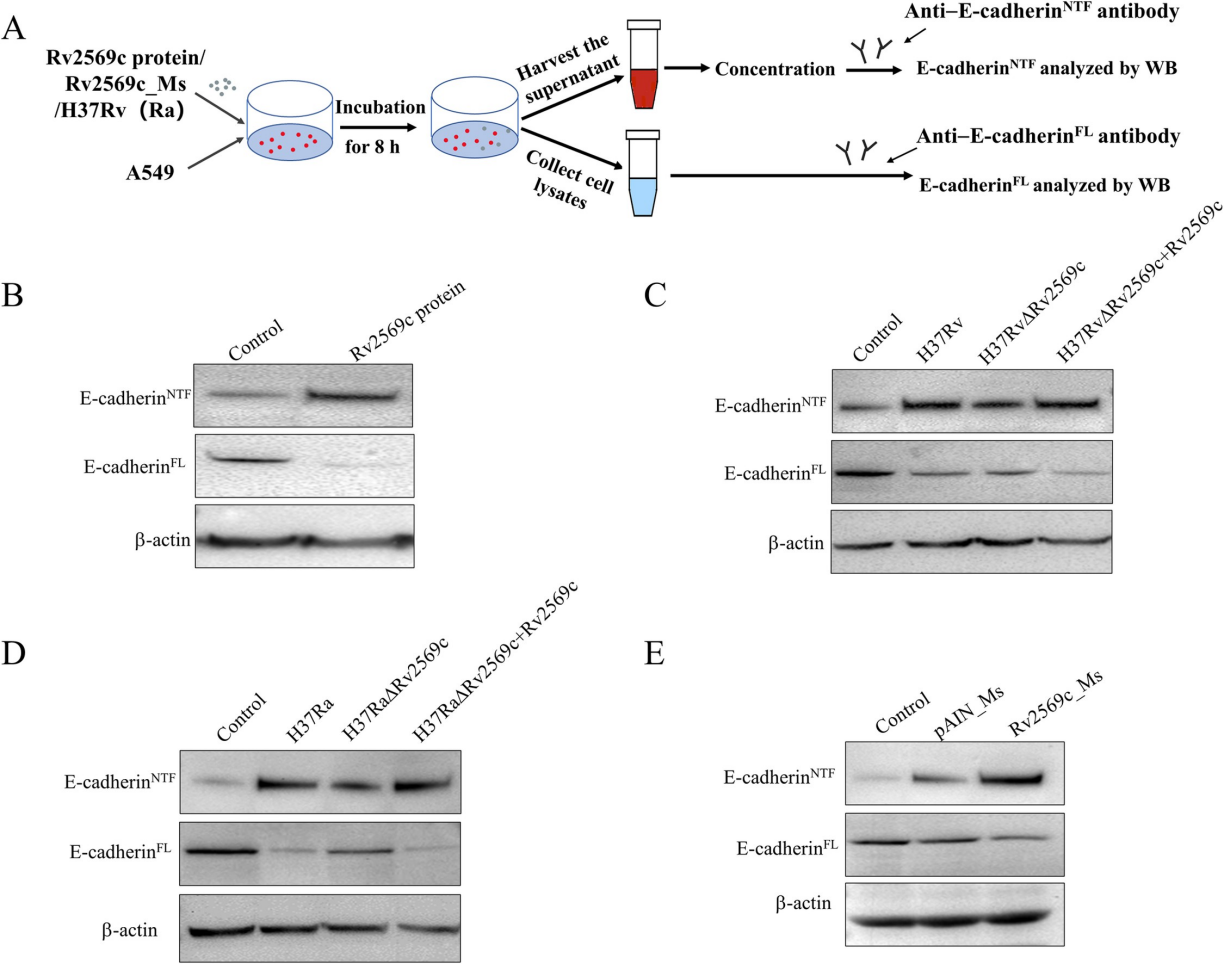
Rv2569c serine protease cleaves E-cadherin of A549 cells *in vitro*. (A) Schematic representation of Rv2569c treatment of A549 cells. After 8 h, concentrated supernatant and cell lysates were analyzed by western blot using an anti–E-cadherin antibody. E-cadherin^FL^: the full-length E-cadherin in the cell lysates; E-cadherin^NTF^: the soluble extracellular E-cadherin fragment. (B) The cleavage of E-cadherin of A549 cells was detected by western blot after treatment with 1 μM Rv2569c protein for 8 h. (C) The cleavage of E-cadherin of A549 cells was detected by western blot after infection with wild-type strain (H37Rv), Rv2569c-deletion mutant (H37RvΔRv2569c), and complementation of H37RvΔRv2569c (H37RvΔRv2569c + Rv2569c) strains (MOI = 10) for 8 h. (D) The cleavage of E-cadherin of A549 cells was detected by western blot after infection with H37Ra, H37RaΔRv2569c, and H37RaΔRv2569c + Rv2569c (MOI = 10) for 8 h. (E) The cleavage of E-cadherin of A549 cells was detected by western blot after infection with pAIN_Ms and Rv2569c_Ms strains (MOI = 10) for 8 h. All assays were repeated three times.

**Fig 4:**
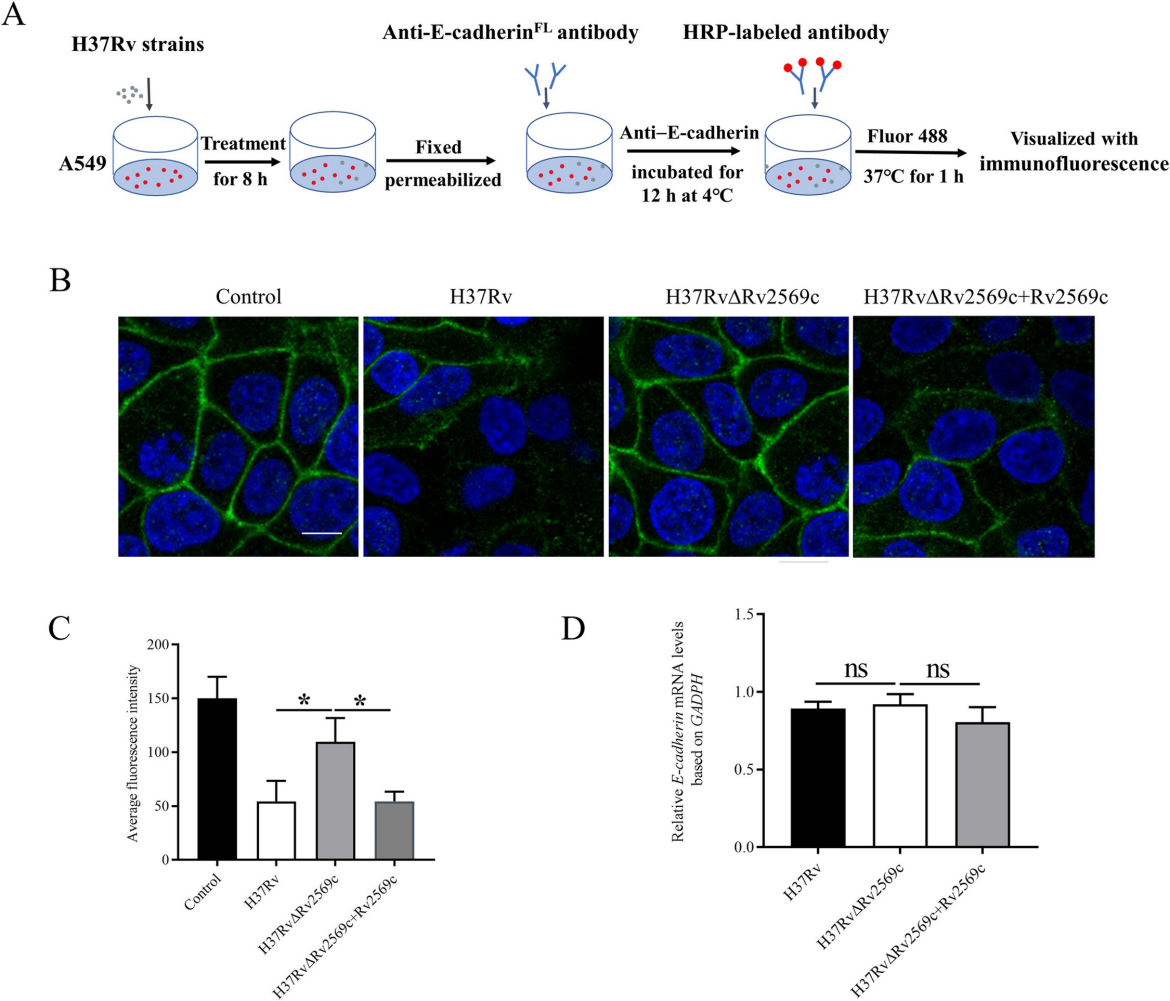
Visualization of E-cadherin degradation by immunofluorescence micrographs *in vitro*. (A) Schematic representation of immunofluorescence observation on A549 cells infected with H37Rv strains. (B) The destruction of E-cadherin was visualized by confocal micrographs with anti–E-cadherin (green) and nuclear staining with DAPI (blue). A549 cells were infected with H37Rv, H37RvΔRv2569c, or H37RvΔRv2569c + Rv2569c for 8 h to observe the destruction of E-cadherin in A549 cells by fluorescence micrographs. Untreated A549 cells were used as the negative control. Scale bars, 10 μm. (C) Total fluorescence intensity of E-cadherin in A549 cells was quantified by Image J (n **=** 100 in triplicate). (D) Relative E-cadherin mRNA levels were measured by qRT-PCR after infection with H37Rv, H37RvΔRv2569c, or H37RvΔRv2569c + Rv2569c strains for 8 h. The error bars represent the SEM of three independent experiments. One-way ANOVA, followed by Bonferroni**’**s multiple-comparison post-hoc test (*P < 0.05, ns: nonsignificant).

To confirm the role of Rv2569c in the cleavage of E-cadherin in epithelial cells, A549 cells were treated with 1 μM purified Rv2569c protein. The levels of the full-length E-cadherin (E-cadherin^FL^) in cell lysates and the extracellular N-terminal fragment of E-cadherin (E-cadherin^NTF^) in cell supernatants were evaluated (Fig 3A). Following treatment with Rv2569c protein, A549 cell monolayers were analyzed for E-cadherin cleavage after 8 h. Western blot analysis demonstrated an increase in the extracellular E-cadherin^NTF^ of approximately 85 kDa in the cell supernatants. In contrast, a reduction in the quantity of E-cadherin^FL^ was found in the cell lysates (Fig 3B). Subsequently, A549 cells were co-cultured with H37Rv/H37Ra, H37Rv/H37RaΔRv2569c, H37Rv/H37RaΔRv2569c + Rv2569c strains, and pAIN_Ms and Rv2569c_Ms strains at multiplicity of infection (MOI) = 10. We found that H37Rv/H37RaΔRv2569c exhibited a decrease in E-cadherin^FL^ in the cell lysates and an increase in E-cadherin^NTF^ in the cell supernatants compared with H37Rv/H37Ra and H37Rv/H37RaΔRv2569c + Rv2569c (Fig 3C and 3D). Additionally, a decrease in the quantity of E-cadherin^FL^ was found after infection with Rv2569c_Ms relative to infection with pAIN_Ms (Fig 3E). Subsequently, immunofluorescence analysis was used to observe the cleavage of E-cadherin using an anti–E-cadherin antibody (Fig 4A). The results of the observation revealed a decrease in the level of E-cadherin protein following the incubation of H37Rv and H37RvΔRv2569c + Rv2569c strains, compared with the H37RvΔRv2569c strain (Fig 4B and 4C). To examine whether the reduction in E-cadherin protein levels was related to a decrease in gene expression, the expression of the E-cadherin gene was analyzed through qRT-PCR. As depicted in Fig 4D, the expression of the E-cadherin gene remained unaltered following incubation with H37Rv, H37RvΔRv2569c, and H37RvΔRv2569c + Rv2569c, indicating that Rv2569c did not exert any influence on the expression of the E-cadherin gene in A549 cells. These results provide evidence that Rv2569c facilitates the cleavage of E-cadherin by *M*.*tb*.

### Rv2569c promotes translocation of *M*.*tb* across the epithelial barrier

**Fig 5:**
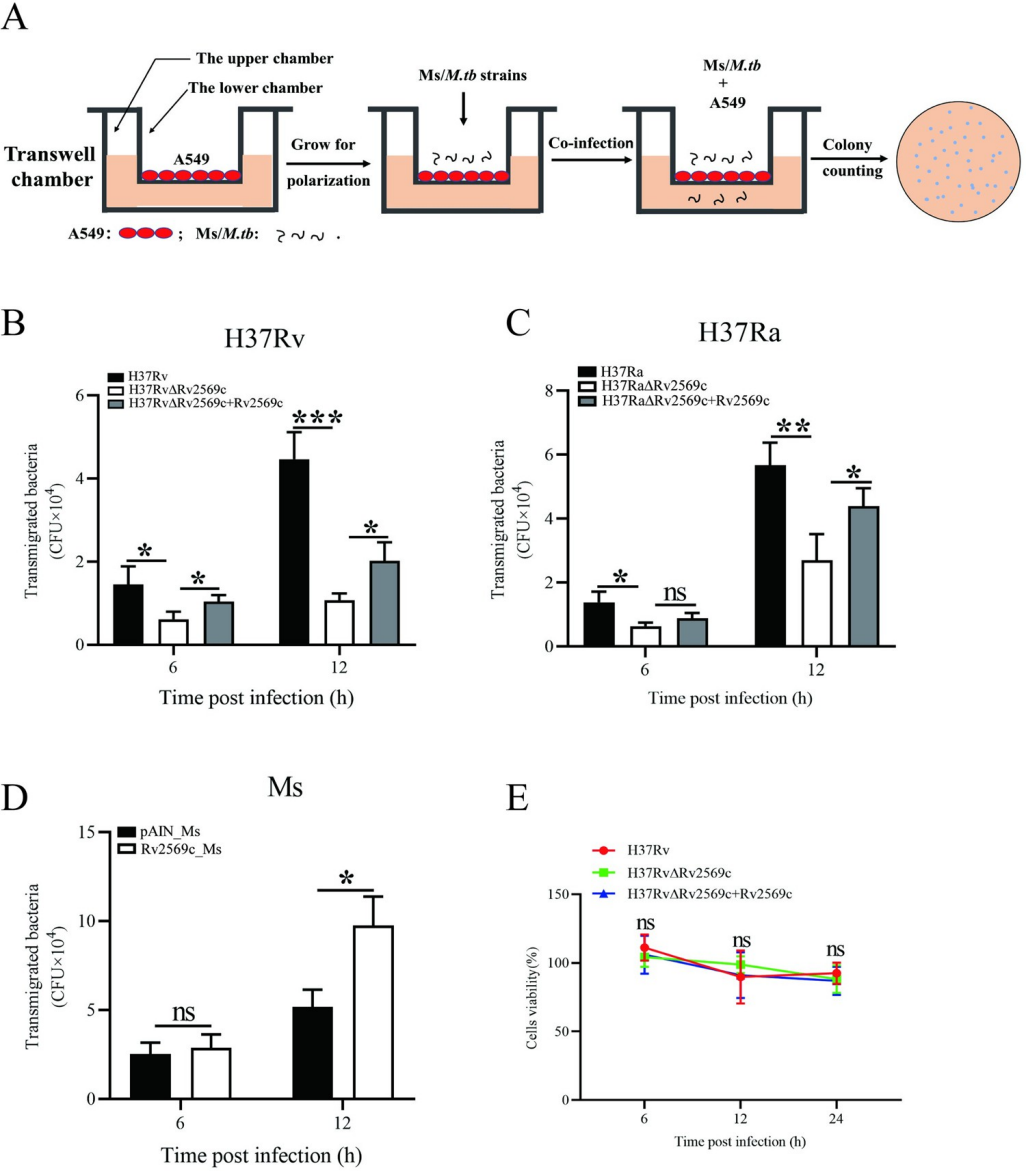
Rv2569c contributes to the translocation of *M*.*tb* from cell to cell. (A) Schematic representation of bacterial translocation is shown. A549 cells were grown to form confluent monolayers and then incubated for another 5 days to allow for cell polarization. Subsequently, they were infected with H37Rv strains (MOI **=** 10), H37Ra strains (MOI **=** 10), or recombinant Ms strains (MOI **=** 10) for 6 and 12 h. Transmigrated bacteria were measured in the lower chamber by counting colony-forming units (CFUs) on 7H10 agar plates. (B) Transmigrated bacteria were evaluated in the lower chamber after infection with H37Rv, H37RvΔRv2569c, and H37RvΔRv2569c + Rv2569c. (C) Transmigrated bacteria were counted in the lower chamber after infection with H37Ra, H37RaΔRv2569c, and H37RaΔRv2569c + Rv2569c. (D) Transmigrated bacteria were counted in the lower chamber after infection with pAIN_Ms and Rv2569c_Ms. (E) Cell viability of A549 cells was evaluated by the CCK-8 assay after infection with H37Rv, H37RvΔRv2569c, and H37RvΔRv2569c + Rv2569c for 6, 12, and 24 h. The error bars represent the SEM of three independent experiments. Two-way ANOVA, followed by Bonferroni**’**s multiple-comparison post-hoc test (*P < 0.05, **P < 0.01, ns: nonsignificant). All assays were repeated three times.

The epithelial barrier plays a crucial role in defending against bacterial invasion [31]. To determine the ability of Rv2569c to traverse the epithelial barrier, a transwell assay was performed using transwell chambers. Following the establishment of cell polarization, A549 cell monolayers were infected with various strains, namely H37Rv/H37RaΔRv2569c + Rv2569c, pAIN_Ms, and Rv2569c_Ms strains (Fig 5A). As anticipated, the bacterial load of H37Rv/H37Ra and H37Rv/H37RaΔRv2569c + Rv2569c was significantly higher than that of H37Rv/H37RaΔRv2569c in the lower chamber (Fig 5B and 5C). Likewise, the bacterial load of Rv2569c_Ms was higher than that of pAIN_Ms (Fig 5D). To investigate whether the bacteria induced cell death during the infection, cell viability was evaluated using CCK-8 (Fig 5E). We found that the presence of Rv2569c did not affect cell proliferation following infection with H37Rv, H37RvΔRv2569c, and H37RvΔRv2569c + Rv2569c strains. These results suggest that Rv2569c promotes the traversal of the epithelial barrier by *M*.*tb* and does not affect cell proliferation.

### Rv2569c promotes colonization by *M*.*tb* and induces pathological injury in mice

**Fig 6:**
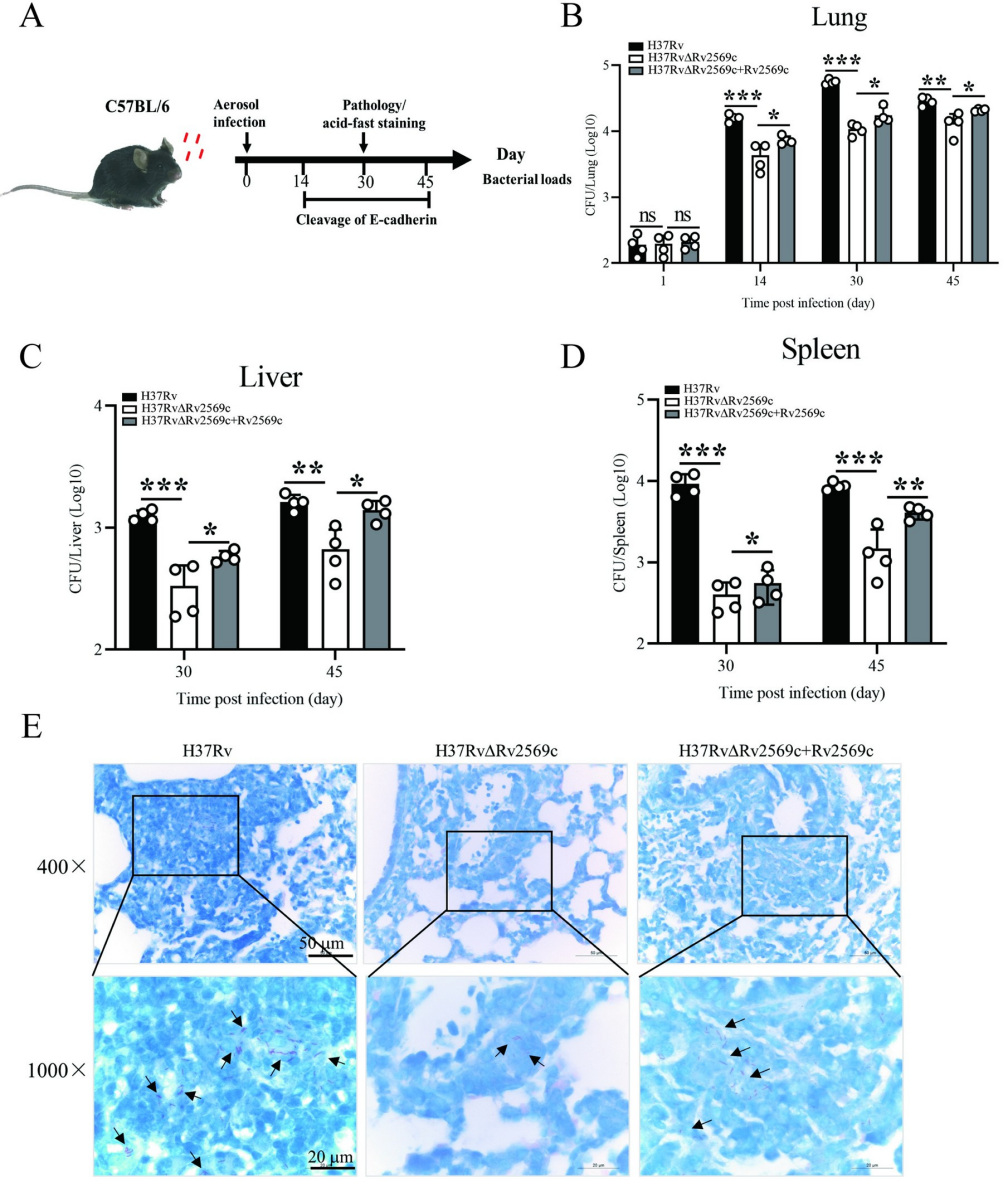
Rv2569c promotes bacterial dissemination *in vivo*. (A) Schematic diagram of H37Rv strains challenge timeline. C57BL/6 mice (n = 4) were aerosol-infected with roughly 200 CFUs of H37Rv, H37RvΔRv2569c, and H37RvΔRv2569c + Rv2569c per mouse for 1 day, 14 days, 30 days, and 45 days. Bacterial load, pathology, and cleavage of E-cadherin were evaluated. (B) Bacterial loads of the lung were evaluated by colony-counting at 1 day, 14 days, 30 days, and 45 days after infection. (C) and (D) Bacterial loads of the liver and spleen in the mice were quantified by colony-counting at 30 days and 45 days after infection. (E) Analysis of acid-fast staining of the lung in the mice at 30 days after infection. The error bars represent the SEM of four independent experiments. Two-way ANOVA, followed by Bonferroni**’**s multiple-comparison post-hoc test (*P < 0.05, **P < 0.01).

**Fig 7:**
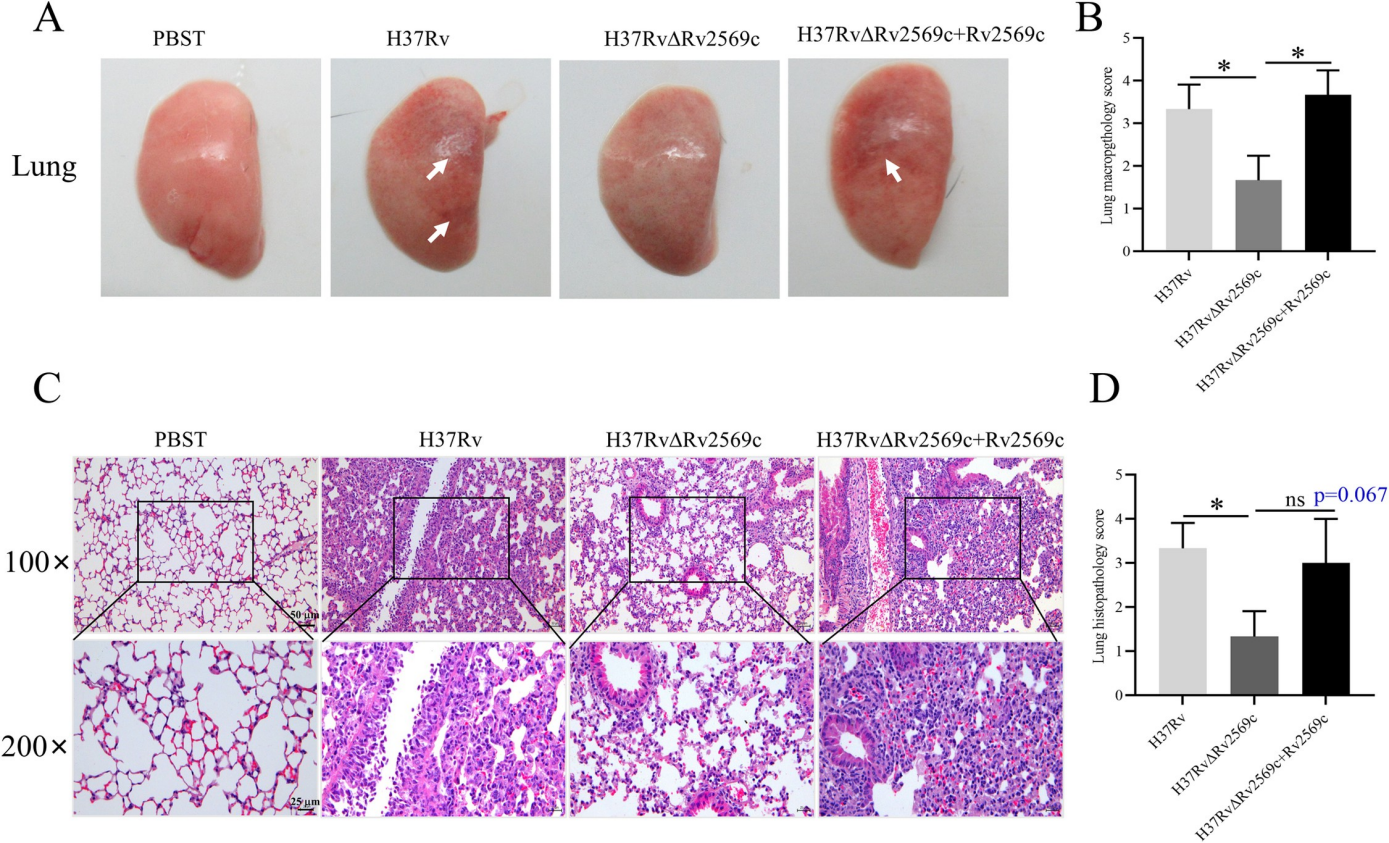
Gross pathology and histopathology of the lungs in mice after challenge with H37Rv, H37RvΔRv2569c, and H37RvΔRv2569c + Rv2569c strains at 30 days after infection. (A) Gross appearance of the lung was examined to observe the severity of lung damage. The lung exhibited more severe bleeding after challenge with H37Rv and H37RvΔRv2569c + Rv2569c strains compared with H37RvΔRv2569c. (B) Gross pathology score of the lung. (C) Histopathology of the lung was conducted to evaluate the pathological damage. There was a greater inflammatory cell infiltration and macrophage aggregation in the lung challenged with H37Rv and H37RvΔRv2569c + Rv2569c strains compared with the lung challenged with H37RvΔRv2569c strains. Data indicate one experiment with 4 independent replicates. (D) Lung histopathology score. The error bars represent the SEM of four independent experiments. One-way ANOVA, followed by Bonferroni**’**s multiple-comparison post-hoc test (ns, nonsignificant; *P < 0.05).

To explore the role of the Rv2569c protein in the pathogenicity of *M*.*tb*, C57BL/6 mice were aerosol-infected with roughly 200 colony-forming units (CFUs) of H37Rv, H37RvΔRv2569c, or H37RvΔRv2569c + Rv2569c strains per mouse for 1 day, 14 days, 30 days, and 45 days. Bacterial burden, pathology, and cleavage of E-cadherin were evaluated (Fig 6A). The results showed that the bacterial burden in the lung infected with H37Rv or H37RvΔRv2569c + Rv2569c was significantly higher than that in the lung infected with H37RvΔRv2569c at 14 days, 30 days, and 45 days but not at 1 day after infection (Fig 6B). Cultivation of bacteria from the liver and spleen failed at 1 day and 14 days after infection. In contrast, at 30 days and 45 days after infection, bacterial load in the liver and spleen infected with H37Rv or H37RvΔRv2569c + Rv2569c was remarkably higher than that in the liver and spleen infected with H37RvΔRv2569c (Fig 6C and 6D). Qualitative analysis using acid-fast staining in the lung yielded similar results at 30 days after challenge (Fig 6E). Subsequently, gross pathology and histopathology were performed to evaluate the severity of lung damage at 30 days after challenge. In terms of gross pathology, the lung exhibited more severe bleeding after challenge with H37Rv or H37RvΔRv2569c + Rv2569c strains relative to H37RvΔRv2569c (Fig 7A and 7B). With regards to histopathology, a large number of inflammatory cells infiltrated and macrophages aggregated in the lung challenged with H37Rv or H37RvΔRv2569c + Rv2569c strains, while the lung challenged with H37RvΔRv2569c demonstrated a marked reduction in inflammatory cell infiltration and macrophage aggregation (Fig 7C and 7D). These results demonstrate that Rv2569c could promote *M*.*tb* dissemination and colonization and induce organ pathological injury *in vivo*.

### Rv2569c is involved in the cleavage of E-cadherin in mice

**Fig 8:**
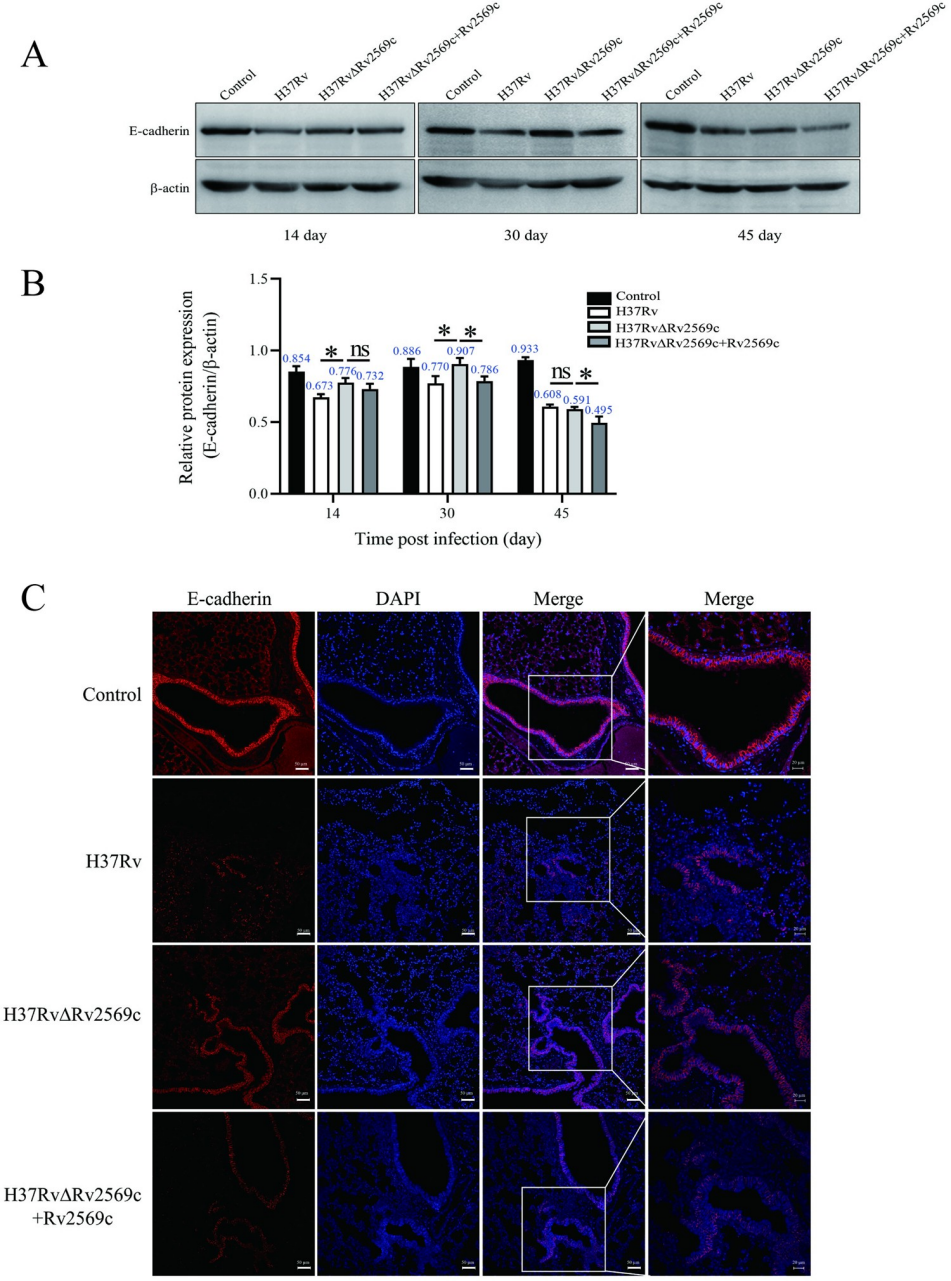
Levels of E-cadherin protein were detected by western blot analysis in the lungs of mice after challenge with the H37Rv strains *in vivo*. (A) Western blot analysis of the expression of E-cadherin in the lung after challenge with H37Rv, H37RvΔRv2569c, and H37RvΔRv2569c + Rv2569c at 14 days, 30 days, and 45 days after infection. (B) Relative protein expression of E-cadherin in the lung based on β-actin measured by Image J. (C) The level of E-cadherin in the lungs was observed by confocal microscopy with anti–E-cadherin (red) and nuclear staining with DAPI (blue) at 30 days after infection. Scale bars, 50 μm. The error bars represent the SEM of three independent experiments. Two-way ANOVA, followed by Bonferroni**’**s multiple-comparison post-hoc test (*P < 0.05).

To assess the impact of the Rv2569c protein on E-cadherin *in vivo*, we conducted western blot and immunofluorescence staining analyses to evaluate the disruption of E-cadherin in the lung tissue of mice following challenge with H37Rv, H37RvΔRv2569c, or H37RvΔRv2569c + Rv2569c strains. The results obtained from the western blot analysis revealed a significant decrease in the level of E-cadherin protein in the lung infected with H37Rv and H37RvΔRv2569c + Rv2569c strains relative to that in the lung infected with H37RvΔRv2569c strain, at 14 and 30 days after infection (Fig 8A and 8B). The tissue immunofluorescence staining assay demonstrated a similar phenomenon at 30 days after infection (Fig 8C). Collectively, these results indicate that Rv2569c contributes to the disruption of the respiratory epithelial barrier *in vivo*.

## Discussion

The alveolar epithelial barrier serves as a critical obstacle for microbial invaders [31,32]. Both TJs and AJs are essential for maintaining the integrity of the epithelial barrier, establishing epithelial polarity, and promoting cell–cell contact [18]. The disruption of the epithelial barrier, either directly or indirectly, is a crucial factor in the ability of many respiratory bacterial pathogens to cause severe systemic infections [33–35]. These pathogens target intercellular junctions through toxins or proteases to traverse the polarized epithelial cell layer. The present investigation provides novel evidence that the serine protease Rv2569c can facilitate the translocation of *M*.*tb* across the epithelial barrier by disrupting E-cadherin, thereby promoting bacterial colonization and inducing pathological injury.

Proteases—a family of enzymes capable of hydrolyzing peptides and proteins—are categorized into five distinct classes, namely serine proteases, cysteine proteases, aspartic proteases, threonine proteases, and metalloproteinases [36,37]. Proteases are known to play crucial roles in the physiological processes of the epithelial barrier, including host-tissue invasion, degradation, and immune-response evasion [37]. The cysteine protease SpeB, produced by *Group* A *Streptococcus*, facilitates translocation across the epithelial barrier by directly cleaving junctional protein E-cadherin [35]. Similarly, the serine protease HtrA produced by *Campylobacter jejuni* impairs the intestinal barrier and invades epithelial cells through the cleavage of occludin [26]. Current research on *M*.*tb* suggests that its dissemination primarily occurs through both the “Trojan horse” and “direct migration” mechanisms [14]. Specifically, while *M*.*tb* may be transported across the epithelial barrier by infected macrophages, *M*.*tb* itself produces adhesins and toxins to directly invade and lyse epithelial cells, thereby crossing the alveolar barrier. *M*.*tb* heparin-binding haemagglutinin adhesin (HBHA) promotes bacterial adherence to alveolar epithelial cells and participates in extrapulmonary dissemination without altering the integrity of tight junctions [38]. In the present study, we reported for the first time the characterization of serine protease Rv2569c and its role in *M*.*tb* translocation across the alveolar epithelial barrier.

According to phylogenetic tree analysis, Rv2569c is widely distributed among mycobacteria, which suggests its essential role in biological function. Notably, E-cadherin, fibrinogen, and fibronectin are implicated in cell adhesion processes [39]. Furthermore, substrate specificity studies have indicated that Rv2569c has the capacity to degrade a variety of protein substrates, thereby potentially contributing to the degradation of cell adhesion. Although the analysis of the conserved domains showed that Rv2569c possessed a cysteine protease activity, our investigation involving the incubation of several inhibitors with casein and E-cadherin revealed that AEBSF and PMSF exhibited an inhibitory activity against Rv2569c. Interestingly, E-64 and N-ethylmaleimide failed to affect the protease activity of Rv2569c, suggesting that Rv2569c is a serine protease rather than a cysteine protease. Serine proteases are critical virulence factors for *M*.*tb* because they are involved in the evasion or subversion of host defenses or tissue degradation, and they represent potential drug targets against tuberculosis [40]. The *M*.*tb* serine protease Rv3194c has been linked to *M*.*tb* pathogenicity, given that it hinders phagocytosis and chemotaxis, thereby promoting *M*.*tb* persistence in the lungs [41]. Additionally, Rv3671c confers protection to *M*.*tb* against acid stress and enhances its ability to withstand phagosome–lysosome fusion [42]. It has been shown that inactivation of HtrA-like serine protease pepD alters the virulence of *M*.*tb* in a mouse model [43].

The role of epithelial barriers as active effectors of microbial defense is being increasingly recognized [31]. E-cadherin, a critical organizer of epithelial cell barriers, is essential for maintaining epithelial tissue integrity and homeostasis [44]. Additionally, E-cadherin is involved in macrophage epithelialization during granuloma formation in *M*.*tb* infection [45]. The destruction of E-cadherin is necessary for bacterial systemic dissemination. Numerous serine proteases produced by pathogenic bacteria contribute to the transmigration of these bacteria across the epithelial barrier and into deeper tissues by cleaving E-cadherin. In addition to cleaving E-cadherin, invasive bacteria employ various strategies to deregulate the expression of the E-cadherin gene and subsequently destroy AJs [46]. This study confirms that Rv2569c is an extracellular protease that directly targets and cleaves E-cadherin, but it does not affect the transcription of E-cadherin during infection. The release of soluble E-cadherin has the potential to initiate an inflammatory response [47]. Furthermore, the reduction of E-cadherin on the surface of epithelial cells can result in the disruption of antimicrobial immune responses [46]. In the case of *M*.*tb* dissemination, ESAT6 can be utilized to induce cytolysis of alveolar epithelial cells, thereby enabling direct dissemination from the alveolar wall [48]. Interestingly, our findings showed that Rv2569c significantly enhanced bacterial transmigration without inducing A549 cell death in the transwell assay. Taken together, these results demonstrate that the proteolytic activity of Rv2569c cleaves E-cadherin, allowing bacterial translocation across the epithelial barrier and promoting systemic dissemination.

Our findings demonstrated that the deletion of Rv2569c from H37Rv resulted in elevated levels of E-cadherin protein in the lungs of infected mice compared with the wild-type H37Rv. This increase in E-cadherin shedding may contribute to the virulence of the bacterial pathogen and is frequently implicated in the development of lung injury [49,50]. It is worth noting that pathogen-encoded extracellular proteases play a crucial role as virulence factors, facilitating pathogen colonization and induction of pathological damage [51,52]. Our study demonstrated that the serine protease Rv2569c promoted the colonization of *M*.*tb* in the lung and facilitated the dissemination of *M*.*tb* to the liver and spleen, exacerbating the pathological damage in the lung of the infected mice.

In summary, our study presents evidence that Rv2569c is an extracellular serine protease of *M*.*tb* that facilitates the cleavage of E-cadherin, thereby enabling the translocation of bacteria across the alveolar epithelial barrier both *in vitro* and *in vivo*. Furthermore, our findings indicate that Rv2569c plays a crucial role in augmenting bacterial burden and inducing pathological injury of the lung in mice. These results provide valuable insights into the pathogenesis mechanisms of *M*.*tb* and may serve as a foundation for the development of innovative therapeutic strategies for TB.

## Materials and methods

### Ethics statement

C57BL/6 female SPF mice (6–8-week-old) were purchased from the Vital River Animal Laboratories (Beijing, China). All of the experimental animals were under the supervision of the Animal Ethics Committee of the Harbin Veterinary Research Institute (Ethical Committee Approval Number: HVRI-IACUC-231116-02-GJ).

### Bacterial strains and culture conditions

*E*. *coli* DH5α and BL21 (DE3) strains were grown in Luria–Bertani (LB) medium for plasmid transformation. *Mycolicibacterium smegmatis* mc^2^155 strain was cultured in Middlebrook 7H9 broth (BD Biosciences, San Jose, CA, USA) containing 0.2% glycerol and 0.05% Tween-80 (Amresco, Solon, OH, USA). *M*.*tb* H37Rv strains (ATCC, 27294) and *M*.*tb* H37Ra strains (ATCC, 25177) were separately grown in Middlebrook 7H9 broth or 7H10 agar (BD Biosciences) supplemented with 0.2% glycerol, 0.05% Tween-80, and 10% oleic acid–albumin–dextrose–catalase (OADC) (BD Biosciences). All of the strains were cultured at 37°C. Kanamycin (50 μg/mL) was added when necessary.

### Expression and purification of Rv2569c

In the present study, we utilized a multistep approach to investigate the function of the Rv2569c protein. First, the conserved domains in the NCBI conserved domain database structure (https://www.ncbi.nlm.nih.gov/Structure/cdd/wrpsb.cgi) were analyzed to predict the function of the protein [30]. Subsequently, MEGA 7 software was employed to perform sequence alignment and phylogenetic tree analysis of the Rv2569c protein using the "Test Neighbor-Joining Tree" method [53]. To obtain the Rv2569c protein, the *rv2569c* gene (GeneBank: NC_000962.3) from *M*.*tb* H37Rv was amplified using specific primers (S1 Table) and cloned into the pET-28a vector. The resulting recombinant plasmid, pET28a-Rv2569c, was transformed into *E*. *coli* BL21 cells, and protein expression was induced by adding IPTG (1 mM). The induced cells were then verified by western blot analysis using an anti–6×His antibody (Proteintech, Wuhan, China). The expressed cells were disrupted by ultrasound in the lysis buffer (20 mM Tris-HCl, 150 mM NaCl, and 5% glycerol; pH 8.0). The Rv2569c protein was subsequently purified using an Ni^2+^ Sepharose 6 Fast Flow column (GE Healthcare, Northampton, MA, USA). To generate an anti-Rv2569c polyclonal antibody, the purified Rv2569c protein was sent to Beijing Biodragon Immunotechnologies Co., Ltd.

### Subcellular location of Rv2569c

Subcellular fractions of *M*.*tb* H37Rv reference strain were prepared as previously described [54]. All of the fractions, including the whole broken cell pellet (CL), cell wall (CW), cell membrane (CM), cytoplasm (CP), and cell culture filtrate (CF), were analyzed via western blot analysis [8]. An anti-Rv2569c antibody (1:3000 dilution) or an anti-Ag85B rabbit polyclonal antibody (1:3000 dilution, Abcam, Cambridge, MA, USA) was used as the primary antibody, and an HRP-labeled goat anti-rabbit antibody was utilized as the secondary antibody (1:10000 dilution, Proteintech). The results were visualized using EZ ECL pico chemiluminescence (Life-iLab, Shanghai, China). Immunoelectron microscopy (IEM) of Rv2569c was performed as described in previous reports from our laboratory [55]. Specifically, *M*.*tb* strains were cultured in 7H9 broth until they reached an OD_600nm_ of 0.8–1.0, which was followed by centrifugation to harvest the cells. Following resuspension and washing with phosphate-buffered saline (PBS), the cellular pellet was fixed with IEM fixative (Servicebio, Wuhan, China) for 2 h at room temperature while being shielded from light. Subsequently, the pellet was subjected to dehydration, embedding, and polymerization to obtain ultrathin sections. These sections were then blocked in 1% bovine serum albumin (BSA) at room temperature for 30 min, followed by incubation with an anti-Rv2569c antibody (1:200 dilution) at 4°C for 12 h. A goat anti-rabbit secondary antibody (1:50 dilution, Sigma-Aldrich) was then used at room temperature for 1 h, after which the samples were stained with 2% uranium acetate–saturated alcohol. Visualization of the samples was carried out using a Hitachi HT7800 transmission electron microscope. The 10-nm black golden particles were considered positive signals.

### Detection of the protease activity of Rv2569c

To demonstrate the activity of Rv2569c protease, a reaction volume (20 mM Tris-HCl, pH 8.0) containing 4 μg of casein and 1 μg of purified Rv2569c protein was incubated for 12 h at 37°C. The negative control consisted of the buffer only. The degradation of casein was analyzed by SDS-polyacrylamide electrophoresis (SDS-PAGE), and casein degradation was analyzed using the Protease Fluorescent Detection Kit (Sigma-Aldrich). Specifically, 1 μg of purified Rv2569c protein was incubated with 20 ng of FITC-casein substrate in the incubation buffer (20 mM Na_3_PO_4_, 150 mM NaCl; pH 7.6) for 1 h at 37°C. Protein buffer and trypsin were employed as negative and positive controls, respectively, and the reaction was terminated by adding a 50% trichloroacetic acid (TCA) solution. The reaction mixture was evaluated using an EnSpire Multiscan Spectrum (PerkinElmer, Waltham, MA, USA) at excitation and emission wavelengths of 485 nm and 535 nm, respectively. All assays were repeated three times.

### Substrate specificity of the Rv2569c protease

To evaluate the substrate specificity of Rv2569c protein, 4 μg of various substrates, including fibrinogen (Novoprotein, Suzhou, China), fibronectin (Novoprotein), and E-cadherin (Novoprotein), was incubated with 1 μg of Rv2569c purified protein in 20 μL of reaction volume (20 mM Tris-HCl, pH 8.0) for 12 h at 37°C. Then, the reaction products were examined by SDS-PAGE.

### Screening of chemical inhibitors of Rv2569c

To assess the protease category of Rv2569c, 1 μg of Rv2569c protein was pretreated with various protease inhibitors, including E-64 (10 μM, Sigma-Aldrich, Shanghai, China), N-ethylmaleimide (1 mM, Sigma-Aldrich), AEBSF (1 mM, Sigma-Aldrich), and PMSF (1 mM, Biosharp, Hefei, China), on ice for 1 h. Subsequently, 4 μg of casein or E-cadherin was added to the reaction system and incubated for 12 h at 37°C, as described above. The results were evaluated by SDS-PAGE.

### Effect of temperature, pH, and divalent cations on the activity of Rv2569c

To evaluate the effect of temperature on Rv2569c protease, 20 μg of casein was incubated with 10 μg of purified Rv2569c protein over a temperature range of 31°C–51°C with intervals of 2°C in a reaction buffer of 200 μL (20 mM Tris-HCl, pH 8.0) for 12 h. The optimal pH was determined at the optimal temperature over a pH gradient of 3.0–11.0 (at 1.0 intervals) for 12 h utilizing Britton–Robinson (20 mM acetic acid, 20 mM boric acid, and 20 mM hydrochloric acid, adjusted to the desired pH using 0.2 M NaOH). Under the conditions of optimal temperature and pH, purified Rv2569c protein (10 μg) was incubated with 5 mM of different divalent cation salts (MgCl_2_, MnCl_2_, BaCl_2_, CaCl_2_, and NiCl_2_) in 200 μL of buffer (20 mM Tris-HCl, pH 8.0) for 12 h. The reaction was terminated by adding a 50% TCA solution, and the resulting reaction products (200 μL) were transferred to a quartz 96-well plate. Quantitative analysis of the results was performed using a Nanophotometer Peal Ultramicro UV-Vis spectrophotometer at 260 nm. One enzymatic activity unit was defined as the amount of enzyme required to increase the absorbance by 0.001 A260 mL^−1^·min^−1^·cm^−1^. All of the assays were repeated three times.

### Construction of Rv2569c-deletion mutant, complementation in H37Rv and H37Ra strains, and recombinant Rv2569c_Ms strains

The Rv2569c-deletion mutant (H37RvΔRv2569c and H37RaΔRv2569c) was generated through phage-mediated specialized transduction, as described in a previous report [56]. Verification of H37RvΔRv2569c and H37RaΔRv2569c was conducted separately using PCR identification and southern blot. The Rv2569c-deletion mutant was then transformed with the recombinant plasmid pMV361-Rv2569c to produce the complemented strains (H37RvΔRv2569c + Rv2569c and H37RaΔRv2569c + Rv2569c), which were also verified using PCR. Additionally, a recombinant plasmid, pAIN-Rv2569c, was constructed and transformed into Ms cells, with positive transformants confirmed through western blot analysis using an anti-Rv2569c antibody. The oligonucleotide primers utilized for amplification in this study are documented in S1 Table. The growth of H37Rv/H37Ra, H37Rv/H37RaΔRv2569c, and H37Rv/H37RaΔRv2569c + Rv2569c was comparatively assessed. Specifically, H37Rv/H37Ra strains were cultured in 100 mL of 7H9 medium following an adjustment to OD_600nm_ = 0.1. The growth curve was measured for 39 days. The colony morphology of the H37Rv/H37RaΔRv2569c strains was compared with that of the H37Rv/H37Ra and H37Rv/H37RaΔRv2569c + Rv2569c strains. The strains were cultivated on 7H10 agar plates to form a single colony, subsequently transferred to another 7H10 plate, and incubated at 37°C for 4–5 weeks to facilitate colony formation. The present study utilized the aforementioned method to measure the growth rate and colony morphology of H37Rv/H37RaΔRv2569c strains and Rv2569c_Ms strains, with all assays repeated in triplicate.

### Rv2569c cleaves E-cadherin of A549 epithelial cells

To investigate the effect of Rv2569c on E-cadherin of A549 cells, the cells were seeded to near confluence in 6-well tissue culture plates with DMEM containing 10% fetal bovine serum (FBS, Ausbian, Sydney, Australia). Prior to treatment, the medium was replaced with fresh serum-free medium. Subsequently, the cells were exposed to 1 μM of purified Rv2569c protein for 8 h at 37°C. Following treatment, the cells were exposed to RIPA lysis buffer (HaiGene, Harbin, China) supplemented with 1% PMSF (Beyotime, Beijing, China) at 4°C for 1 h. The samples were centrifuged at 12000 *g* at 4°C for 10 min to obtain the supernatant, which was subsequently used to analyze the full-length E-cadherin in the cell lysates via western blot analysis. The soluble extracellular E-cadherin fragment was detected in the collected cell-culture medium. The cell lysates and the medium were electrophoresed in SDS-PAGE gels and transferred onto PVDF membranes. The membranes were then blocked with 5% skimmed milk in PBS at 37°C for 2 h, followed by incubation with anti–E-cadherin^FL^ (1:1000 dilution, CST, Danvers, MA, USA), anti–E-cadherin^NTF^ (1:1000 dilution, ABclonal, Wuhan, China), and anti–β-actin (1:1000, CST) antibodies at 4°C overnight. An HRP-labeled goat anti-rabbit antibody was utilized as the secondary antibody. The results were visualized using EZ ECL pico chemiluminescence, and the assays were conducted in triplicate.

For the infection assay, A549 cells were co-cultured with H37Rv, H37RvΔRv2569c, and H37RvΔRv2569c + Rv2569c (MOI = 10), or with H37Ra, H37RaΔRv2569c, and H37RaΔRv2569c + Rv2569c (MOI = 10), or with pAIN_Ms and Rv2569c_Ms (MOI = 10), at 37°C for 8 h. Following infection, western blot analysis was performed on the cell lysates and medium, as previously described.

For immunofluorescence staining, a confluent monolayer of A549 cells was cultured in confocal dishes (Sigma-Aldrich) and subsequently infected with H37Rv, H37RvΔRv2569, or H37RvΔRv2569c+Rv2569c for 8 h. Following infection, the cells were washed thrice with PBS and fixed with 4% paraformaldehyde at room temperature for 20 min. First, A549 cells were permeabilized using 0.1% (v/v) Triton X-100 in PBS for 20 min. Next, they were blocked with 5% (w/v) BSA in PBS at room temperature for 1 h and subsequently incubated with an anti–E-cadherin^FL^ antibody (1:100 dilution, CST) in BSA at 4°C overnight. The cells were then treated with Alexa Fluor 488 goat anti-rabbit IgG (1:500 dilution, Invitrogen, CA, USA) for 1 h and stained with 5 μg/mL DAPI (Invitrogen) for 10 min at room temperature. Finally, the dishes were mounted with 50% glycerol and visualized using confocal laser scanning microscopy (Carl Zeiss, Jena, Germany). The average fluorescence intensity of 100 cells was measured using Image J to quantify the levels of E-cadherin protein.

### qRT-PCR for E-cadherin expression

A549 cells were infected with H37Rv, H37RvΔRv2569c, or H37RvΔRv2569c + Rv2569c (MOI = 10), at 37°C for 8 h, and total RNA was then extracted from the cells using Trizol reagent (ThermoFisher Scientific, Waltham, GA, USA). The expression of E-cadherin in A549 cells was assessed quantitatively by qRT-PCR. The primer sequences utilized for expression analysis were as follows: forward primer GAAGCTGGCTGACATGTACG and reverse primer CTCAAGGGAAGGGAGCTGAA for human E-cadherin, and forward primer AGGTCGGAGTCAACGGATTT and reverse primer ATGAAGGGGTCATTGATGGCA for human GAPDH, which was employed as an internal reference.

### Bacterial transmigration assay

The transwell assay was performed using a 24-well Transwell chamber with a 5-μm pore size (Corning, New York, NY, USA). A549 cells were seeded onto the upper chamber at a density of 3×10^4^ cells/well, while the lower chamber was filled with 500 μL DMEM containing 10% FBS. The cells were allowed to grow to confluent monolayers and then incubated for another 5 days to allow cell polarization. Subsequently, the cells were infected with H37Rv, H37RvΔRv2569c, or H37RvΔRv2569c + Rv2569c (MOI = 10), or with H37Ra, H37RaΔRv2569c, or H37RaΔRv2569c + Rv2569c (MOI = 10), or with pAIN_Ms and Rv2569c_Ms (MOI = 10), at 37°C for 6 and 12 h. Following infection, CFUs were counted to quantify the loads of transmigrated bacteria in the lower chamber on 7H10 agar plates. All assays were performed three times.

### Cell proliferation assay

A549 cells were seeded in 96-well plates with DMEM containing 10% FBS at a density of 2000 cells/well and cultured for 12 h. Subsequently, the cells were infected with H37Rv, H37RvΔRv2569c, or H37RvΔRv2569c + Rv2569c (MOI = 10) for 6, 12, and 24 h. Then, 10 μL of CCK-8 (Beyotime, Shanghai, China) was added to each well and incubated for 1 h. The absorbance was measured at 450 nm to determine the number of viable cells in each well. Cell viability (%) was calculated using the formula: (Treatment group − Blank group)/(Control group − Blank group) × 100%.

### Mouse infection

Female C57BL/6 mice aged 6–8 weeks (n = 4 per group) were subjected to aerosol-infection with H37Rv, H37RvΔRv2569c, or H37RvΔRv2569c+Rv2569c. Prior to infection, the *M*.*tb* strains (OD_600nm_ = 1.0) were subjected to three washes with phosphate buffered saline contained 0.05% Tween-20 (PBST). Each mouse was made to inhale approximately 200 CFUs of the appropriate strains using an aerosol infection chamber at the Biosafety Level-3 (BSL-3) Laboratory [57,58]. The mice were euthanized at 1 day, 14 days, 30 days, and 45 days after infection. The left lung, liver, and spleen were removed, photographed, and homogenized with PBST. Serial dilutions were made onto 7H10 plates and incubated at 37°C for 3–4 weeks to evaluate the bacterial load by counting the CFUs. A right-lung section was subjected to homogenization using RIPA lysis buffer supplemented with PMSF and protease inhibitor cocktail (Roche, Shanghai, China) to obtain the total protein. The E-cadherin protein levels were analyzed by western blot using the total protein at 14 days, 30 days, and 45 days after infection. Another right-lung section was fixed with 4% neutral buffered formalin, embedded in paraffin blocks, and sectioned at 4 μm for acid-fast staining, hematoxylin and eosin (H&E) staining, and immunofluorescence staining. The H&E staining was employed to assess the lung tissue histopathology. A double-blind evaluation of lung injury scoring was performed by two independent pathologists. For gross pathology scoring, the percentage of bleeding area on the lung surface was scored from 0 to 4 as follows: 0 = normal (no bleeding); 1 = mild bleeding (< 30%); 2 = moderate bleeding (≥ 30% and < 50%); 3 = marked bleeding (≥ 50% and < 80%); 4 = severe bleeding (≥ 80%). For histopathology scoring, the extent of lung lesion was scored from 0 to 4 as follows: 0 = normal (no lesion); 1 = slight inflammatory cell infiltration (< 30%); 2 = moderate inflammatory cell infiltration (≥ 30 and < 50%); 3 = severe inflammatory cell infiltration (≥ 50% and < 80%); 4 = severe inflammatory cell infiltration and local macrophage proliferation (≥ 80%). Qualitative detection of bacterial burden in the lung sections was performed through acid-fast staining using a commercial Ziehl–Neelsen stain kit (Baso, Zhuhai, China). The immunofluorescence assay involved dewaxing and antigen repair of the sections, followed by blocking with 3% BSA in PBS at room temperature for 30 min. Subsequently, the sections were incubated with an anti–E-cadherin^FL^ antibody (1:200 dilution) at 4°C overnight and treated with Alexa Fluor 594 goat anti-rabbit IgG (1:400 dilution, Invitrogen, CA, USA) for 1 h. After washing, the samples were stained with 5 μg/mL DAPI at room temperature for 10 min, and the cells were visualized under a confocal laser scanning microscope. The assay was repeated in triplicate.

### Statistical analysis

Data shown as the mean ± standard error of the mean (SEM) were analyzed using GraphPad Prism software (version 9.0; GraphPad, San Diego, CA, USA). Statistical significance was determined by two-way ANOVA, followed by Bonferroni’s multiple-comparison test. The values of n represent the number of mice in the experiments. *P* value < 0.05 was considered statistically significant (*P < 0.05; **P < 0.01; ***P < 0.001).

## Supporting information

S1 Fig Identification of the Rv2569c-deletion mutant. (A) Schematic of the construction of the Rv2569c-deletion mutant. *Pte* I and *Nco* I restriction sites and probe locations of southern blot and the primers used for PCR identification are indicated. (B) PCR identification of H37Rv and H37RvΔRv2569c. (C) Southern blot of genomic DNA from H37Rv and H37RvΔRv2569c digested with *Pte* I and *Nco* I. (D) PCR identification of H37Ra and H37RaΔRv2569c. (E) Southern blot of H37Ra and H37RaΔRv2569c digested with *Pte* I and *Nco* I.
(TIF)

S2 Fig Construction of the complementation of H37Rv/H37RaΔRv2569c with Rv2569c and the expression of Rv2569c in *Mycolicibacterium smegmatis*. (A) PCR identification of complementation of H37Ra/H37RvΔRv2569c with Rv2569c. M: DL2000 marker; 1: PCR identification of H37RaΔRv2569c+Rv2569c; 2: PCR identification of H37RvΔRv2569c + Rv2569c. (B) Identification of pAIN_Ms and Rv2569c_Ms by western blot using anti-Rv2569c antibody.
(TIF)

S3 Fig Effect of Rv2569c on the growth and colony morphology of mycobacteria strains. (A) Growth rate of H37Rv, H37RvΔRv2569c, and H37RvΔRv2569c + Rv2569c. (B) Colony morphology of H37Rv, H37RvΔRv2569c, and H37RvΔRv2569c + Rv2569c. (C) Growth rate of H37Ra, H37RaΔRv2569c, and H37RaΔRv2569c + Rv2569c. (D) Colony morphology of H37Ra, H37RaΔRv2569c, and H37RaΔRv2569c + Rv2569c. (E) Growth rate of pAIN_Ms and Rv2569c_Ms. (F) Colony morphology of pAIN_Ms and Rv2569c_Ms.
(TIF)

S1 Table The complementary mutagenic oligonucleotides used in this study. (DOCX)

S2 Table Effect of different divalent cations on Rv2569c activity. (DOCX)

S1 Data Raw data that underlies this paper. (DOCX)
