# Supplementary figures and images for: Serine protease Rv2569c facilitates transmission of Mycobacterium tuberculosis via disrupting the epithelial barrier by cleaving E-cadherin
Source: PLoS Pathog. 2024 May 9;20(5):e1012214. doi: 10.1371/journal.ppat.1012214 (PMC11081392; doi:10.1371/journal.ppat.1012214)

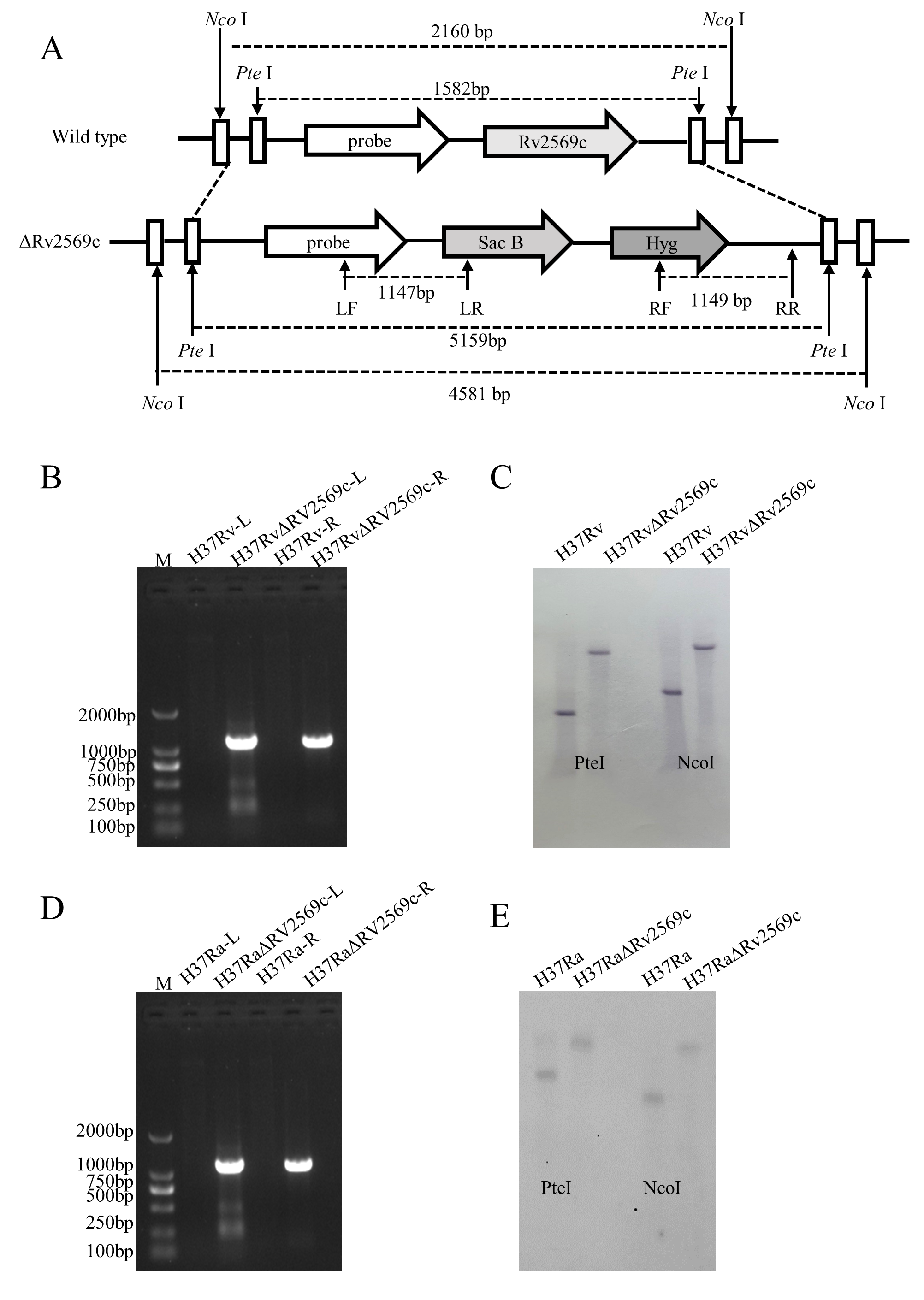

Supplement: S1 Fig — (A) Schematic of the construction of the Rv2569c-deletion mutant. Pte I and Nco I restriction sites and probe locations of southern blot and the primers used for PCR identification are indicated. (B) PCR identification of H37Rv and H37RvΔRv2569c. (C) Southern blot of genomic DNA from H37Rv and H37RvΔRv2569c digested with Pte I and Nco I. (D) PCR identification of H37Ra and H37RaΔRv2569c. (E) Southern blot of H37Ra and H37RaΔRv2569c digested with Pte I and Nco I. (TIF) [file ppat.1012214.s001.tif]

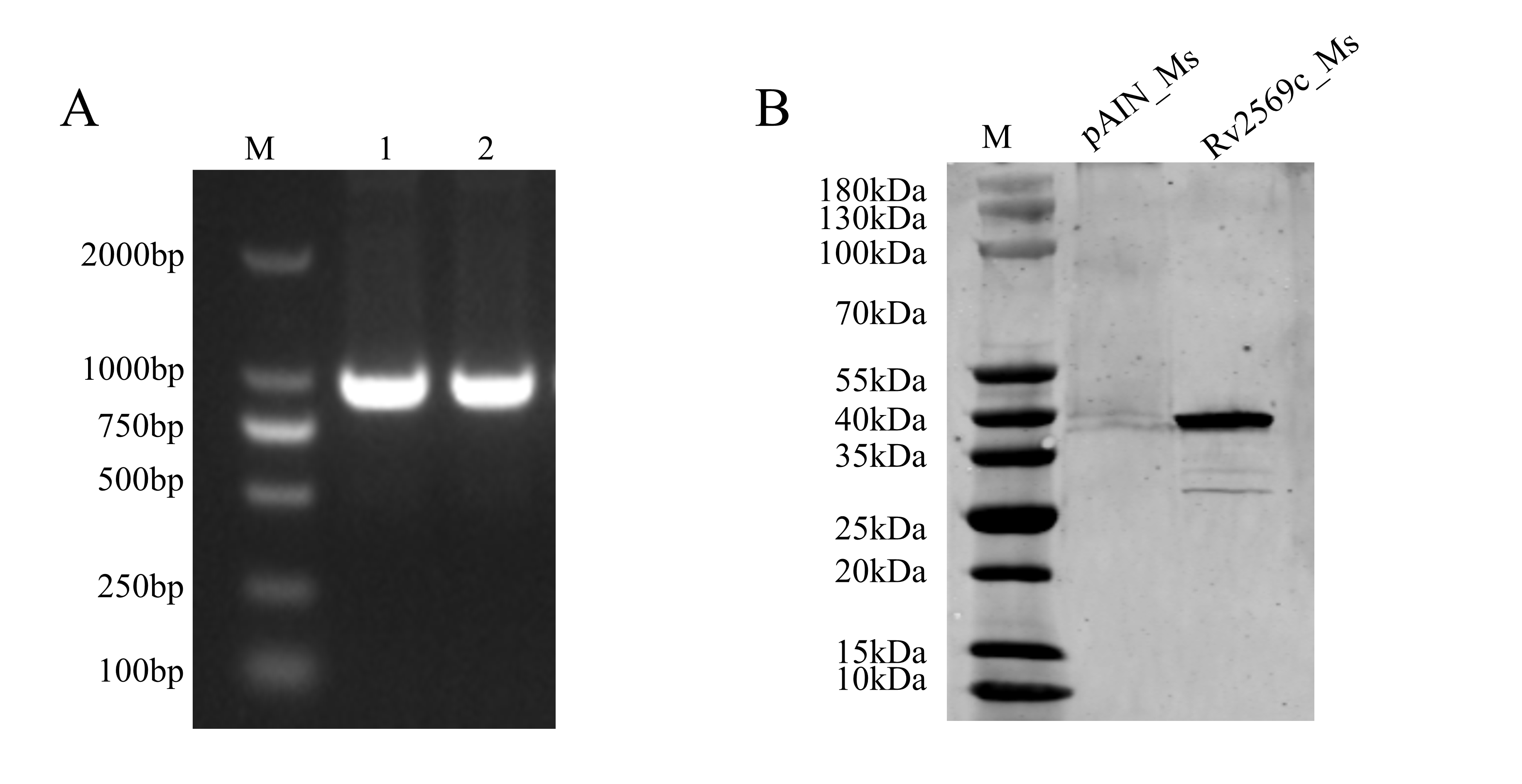

Supplement: S2 Fig — (A) PCR identification of complementation of H37Ra/H37RvΔRv2569c with Rv2569c. M: DL2000 marker; 1: PCR identification of H37RaΔRv2569c+Rv2569c; 2: PCR identification of H37RvΔRv2569c + Rv2569c. (B) Identification of pAIN_Ms and Rv2569c_Ms by western blot using anti-Rv2569c antibody. (TIF) [file ppat.1012214.s002.tif]

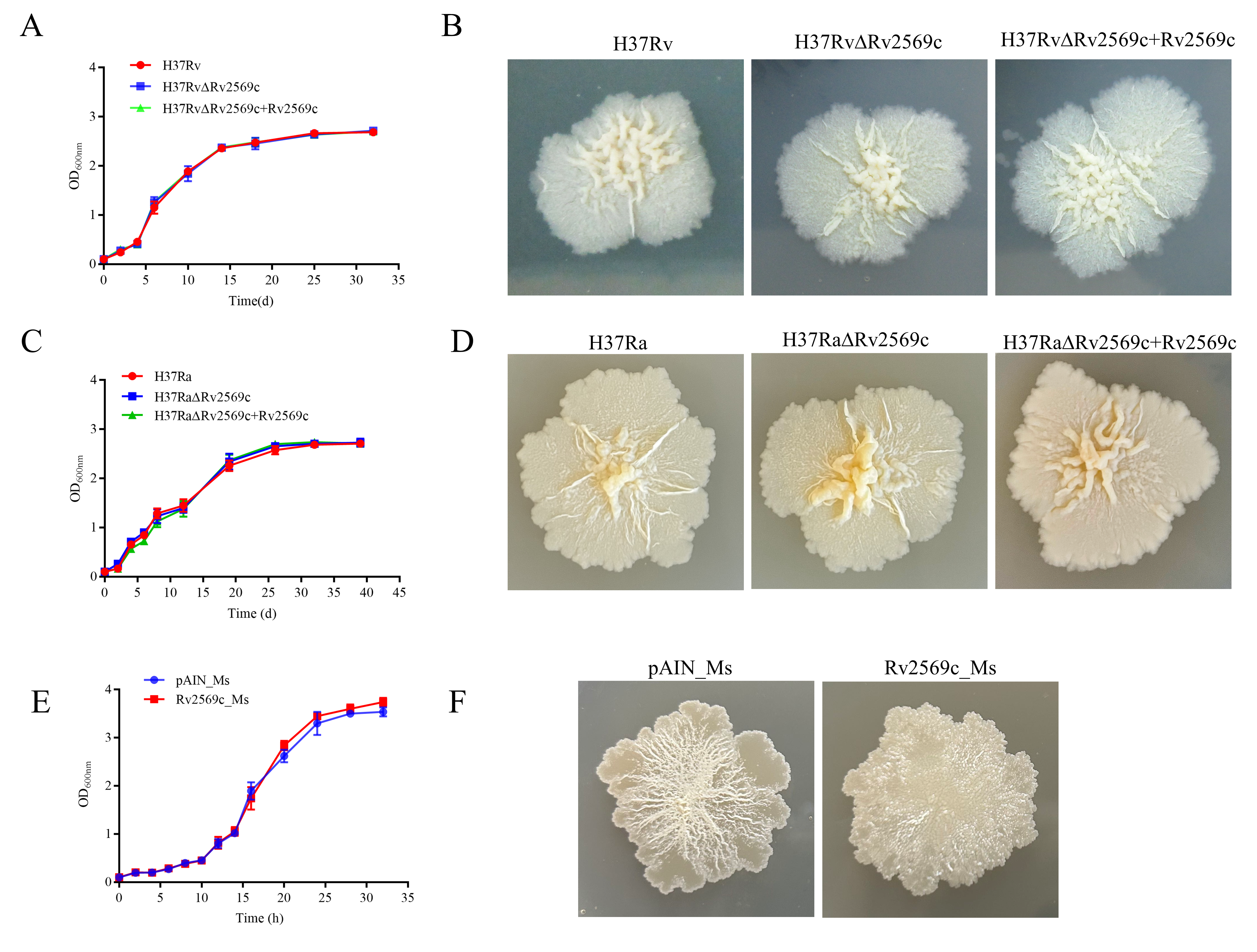

Supplement: S3 Fig — (A) Growth rate of H37Rv, H37RvΔRv2569c, and H37RvΔRv2569c + Rv2569c. (B) Colony morphology of H37Rv, H37RvΔRv2569c, and H37RvΔRv2569c + Rv2569c. (C) Growth rate of H37Ra, H37RaΔRv2569c, and H37RaΔRv2569c + Rv2569c. (D) Colony morphology of H37Ra, H37RaΔRv2569c, and H37RaΔRv2569c + Rv2569c. (E) Growth rate of pAIN_Ms and Rv2569c_Ms. (F) Colony morphology of pAIN_Ms and Rv2569c_Ms. (TIF) [file ppat.1012214.s003.tif]
