# Supplementary material for: Serine protease Rv2569c facilitates transmission of Mycobacterium tuberculosis via disrupting the epithelial barrier by cleaving E-cadherin
Source: PLoS Pathog. 2024 May 9;20(5):e1012214. doi: 10.1371/journal.ppat.1012214 (PMC11081392; doi:10.1371/journal.ppat.1012214)
Supplement: S1 Table — (DOCX) [file ppat.1012214.s004.docx]

**S1 Table. The complementary mutagenic oligonucleotides used in this study**

| Primer | Sequence 5’ −3’ |
| --- | --- |
| Rv2569c-F  Rv2569c-R | AATGGGTCGCGGATCCGAATTCTTGTCAGCAGATAGCTCGTTGTC  TGCGGCCGCAAGCTTCGCATGCAGCGCATC |
| LFP | TTTTTTTTGCCTAAATGGCGCTGGTCAGTTCGGTGTTGC |
| LRP  RFP  RRP  LYZFP  LYZRP  RYZFP  RYZRP  Rv2569c (com)-F  Rv2569c (com)-R  Rv2569c(com) ′-F  Rv2569c(com) ′-R | TTTTTTTTGCCTTTCTGGCCGGTATTCGGTGCGGTGT  TTTTTTTTGCCTAGATGGCGAGTGGCTGGGGCTGGAT  TTTTTTTTGCCTCTTTGGCCGGGACAGATAGTCGCTTGA  GATCGACTGCATCAGCAGCTGA  GTGGACCTCGACGACCCTAG  TGGATCTCTCCGGCTTCACC  TGCAGGTAATGAGCGAACGTCT  GATCCAGCTGCAGAATTCTTGTCAGCAGATAGCTCGTT  GTCGACATCGATAAGCTTTCACGCATGCAGCGCATC  GTGGCAGCGAGGACAACTTG  CCCGACGTCAGGTGGCTAG |
