# Supplementary material for: Serine protease Rv2569c facilitates transmission of Mycobacterium tuberculosis via disrupting the epithelial barrier by cleaving E-cadherin
Source: PLoS Pathog. 2024 May 9;20(5):e1012214. doi: 10.1371/journal.ppat.1012214 (PMC11081392; doi:10.1371/journal.ppat.1012214)
Supplement: S2 Table — (DOCX) [file ppat.1012214.s005.docx]

**S2 Table. Effect of different divalent cations on Rv2569c activity**

| Divalent cation (5 mM) | Relative activity (%) |
| --- | --- |
| None  MgCl_2_  MnCl_2_  BaCl_2_  CaCl_2_  NiCl_2_ | 100±3.55  117.3±1.77  107.4±4.47  101.8±6.31  100.3±6.67  94.7±1.00 |

The errors are represented as standard deviation.
