# Supplementary material for: Serine protease Rv2569c facilitates transmission of Mycobacterium tuberculosis via disrupting the epithelial barrier by cleaving E-cadherin
Source: PLoS Pathog. 2024 May 9;20(5):e1012214. doi: 10.1371/journal.ppat.1012214 (PMC11081392; doi:10.1371/journal.ppat.1012214)
Supplement: S1 Data — (DOCX) [file ppat.1012214.s006.docx]

Raw data that underlies this paper

Fig 2C: Quantitative analysis of FITC-labeled casein digestion by Rv2569c protein

Fluorescence Units [485/530]

| Casein | Casein+Rv2569c | Rv2569c |
| --- | --- | --- |
| 7432 | 97053 | 45632 |
| 7664 | 99255 | 52244 |
| 7852 | 94538 | 47389 |

Fig 2F The effect of temperature on the activity of Rv2569c protease was analyzed quantitatively by spectrophotometry

A260 value

| Control | 31℃ | 33℃ | 35℃ | 37℃ | 39℃ | 41℃ | 43℃ | 45℃ | 47℃ | 49℃ | 51℃ |
| --- | --- | --- | --- | --- | --- | --- | --- | --- | --- | --- | --- |
| 0.243 | 0.254 | 0.261 | 0.256 | 0.279 | 0.263 | 0.265 | 0.252 | 0.246 | 0.251 | 0.244 | 0.241 |
| 0.239 | 0.244 | 0.251 | 0.262 | 0.292 | 0.275 | 0.263 | 0.249 | 0.244 | 0.241 | 0.241 | 0.24 |
| 0.23 | 0.242 | 0.249 | 0.269 | 0.289 | 0.273 | 0.258 | 0.254 | 0.247 | 0.241 | 0.249 | 0.238 |

Fig 2G The effect of pH on the activity of Rv2569c protease was analyzed quantitatively by spectrophotometry.

A260 value

| Control | 3 | 4 | 5 | 6 | 7 | 8 | 9 | 10 | 11 |
| --- | --- | --- | --- | --- | --- | --- | --- | --- | --- |
| 0.13 | 0.77 | 1.34 | 3.98 | 4.46 | 6.29 | 7.06 | 12.16 | 4.06 | 0.56 |
| 0.15 | 0.66 | 1.88 | 6.06 | 6.56 | 6.76 | 8.76 | 14.76 | 3.76 | 1.70 |
| 0.2 | 1.26 | 1.76 | 3.62 | 5.30 | 6.06 | 4.96 | 10.16 | 4.36 | 1.34 |

Fig 3B, C, D and E Cleavage of E-cadherin of A549 cells was detected by western blot


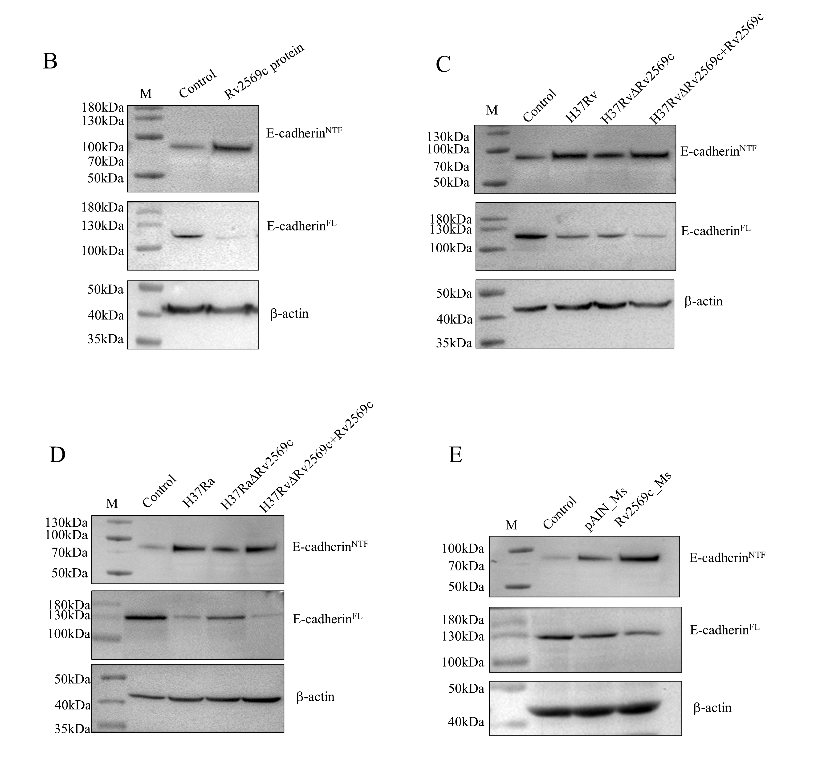


Fig 4C Total fluorescence intensity of E-cadherin in A549 cells was quantified by ImageJ

Average fluorescence intensity

| Control | H37Rv | H37RvΔRv2569c | H37RvΔRv2569c+Rv2569c |
| --- | --- | --- | --- |
| 130 | 35 | 100 | 63 |
| 150 | 55 | 94 | 55 |
| 170 | 73 | 135 | 45 |

Fig 4D Relative E-cadherin mRNA levels were measured by qRT-PCR after infection with H37Rv, H37RvΔRv2569c, or H37RvΔRv2569c + Rv2569c strains for 8 h.

Ct值（*E-cadherin*/*GAPDH*）

| Control | H37Rv | H37RvΔRv2569c | H37RvΔRv2569c+Rv2569c |
| --- | --- | --- | --- |
| 30.349/19.156 | 29.78/18.136 | 28.360/16.895 | 29.388/18.032 |
| 29.7/18.023 | 28.949/17.441 | 28.801/17.337 | 30.279/18.604 |
| 29.34/18.006 | 28.844/17.280 | 28.872/17.218 | 30.268/18.696 |

Fig 5B Transmigrated bacteria were evaluated in the lower chamber after infection with H37Rv, H37RvΔRv2569c, and H37RvΔRv2569c + Rv2569c.

6 h (CFUs)

| H37Rv | H37RvΔRv2569c | H37RvΔRv2569c+Rv2569c |
| --- | --- | --- |
| 19000 | 4200 | 12100 |
| 10300 | 7900 | 10200 |
| 14300 | 6300 | 9000 |

12 h (CFUs)

| H37Rv | H37RvΔRv2569c | H37RvΔRv2569c+Rv2569c |
| --- | --- | --- |
| 45000 | 9300 | 25300 |
| 51000 | 12500 | 17000 |
| 38000 | 10400 | 18400 |

Fig 5C Transmigrated bacteria were counted in the lower chamber after infection with H37Ra, H37RaΔRv2569c, and H37RaΔRv2569c + Rv2569c.

6 h (CFUs)

| H37Ra | H37RaΔRv2569c | H37RaΔRv2569c+Rv2569c |
| --- | --- | --- |
| 16900 | 6700 | 7000 |
| 10300 | 5100 | 10300 |
| 14300 | 7300 | 9100 |

12 h (CFUs)

| H37Ra | H37RaΔRv2569c | H37RaΔRv2569c+Rv2569c |
| --- | --- | --- |
| 58000 | 18000 | 39000 |
| 49000 | 29000 | 43000 |
| 63000 | 34000 | 50000 |

Fig 5D Transmigrated bacteria were counted in the lower chamber after infection with pAIN_Ms and Rv2569c_Ms

6 h (CFUs)

| pAIN_Ms | Rv2569c_Ms |
| --- | --- |
| 20600 | 29300 |
| 23000 | 35900 |
| 32500 | 20700 |

12 h (CFUs)

| pAIN_Ms | Rv2569c_Ms |
| --- | --- |
| 43000 | 99500 |
| 62000 | 112800 |
| 50500 | 80400 |

Fig 5E Cell viability of A549 cells was evaluated by the CCK-8 assay after infection with H37Rv, H37RvΔRv2569c, and H37RvΔRv2569c + Rv2569c for 6, 12, and 24 h.

6 h (OD450 value)

| Control | H37Rv | H37RvΔRv2569c | H37RvΔRv2569c+Rv2569c |
| --- | --- | --- | --- |
| 0.945 | 1.033 | 0.96 | 0.877 |
| 0.966 | 1.005 | 0.97 | 1.048 |
| 0.977 | 1.175 | 1.082 | 1.139 |

12 h (OD450)

| Control | H37Rv | H37RvΔRv2569c | H37RvΔRv2569c+Rv2569c |
| --- | --- | --- | --- |
| 0.664 | 0.717 | 0.672 | 0.704 |
| 0.695 | 0.514 | 0.63 | 0.504 |
| 0.56 | 0.493 | 0.594 | 0.54 |

24 h (OD450)

| Control | H37Rv | H37RvΔRv2569c | H37RvΔRv2569c+Rv2569c |
| --- | --- | --- | --- |
| 0.911 | 0.886 | 0.815 | 0.803 |
| 0.918 | 0.868 | 0.7 | 0.87 |
| 0.886 | 0.758 | 0.88 | 0.687 |

Fig 6B Bacterial loads of the lung were evaluated by colony-counting at 1 day, 14 days, 30 days, and 45 days after infection.

1 d (CFUs)

| H37Rv | H37RvΔRv2569c | H37RvΔRv2569c+Rv2569c |
| --- | --- | --- |
| 200  160 | 160  120 | 250  240 |
| 120 | 260 | 160 |
| 280 | 240 | 180 |

14 d (CFUs)

| H37Rv | H37RvΔRv2569c | H37RvΔRv2569c+Rv2569c( |
| --- | --- | --- |
| 16000 | 5960 | 8880 |
| 18400 | 6000 | 7200 |
| 16000  13200 | 3080  2280 | 6400  6800 |

30 d (CFUs)

| H37Rv | H37RvΔRv2569c | H37RvΔRv2569c+Rv2569c |
| --- | --- | --- |
| 51200  62400 | 8000  12800 | 13480  14800 |
| 53600 | 10000 | 16000 |
| 56800 | 12000 | 25520 |

45 d (CFUs)

| H37Rv | H37RvΔRv2569c | H37RvΔRv2569c+Rv2569c |
| --- | --- | --- |
| 31600  32400 | 14000  7200 | 21840  19840 |
| 27200 | 143200 | 20120 |
| 23200 | 186800 | 21600 |

Fig 6C Bacterial loads of the liver in the mice were quantified by colony-counting at 30 days and 45 days after infection

30 d (CFUs)

| H37Rv | H37RvΔRv2569c | H37RvΔRv2569c+Rv2569c |
| --- | --- | --- |
| 1147 | 187 | 587 |
| 1413 | 483 | 520 |
| 1307 | 453 | 547 |
| 1147 | 207 | 667 |

45 d (CFUs)

| H37Rv | H37RvΔRv2569c | H37RvΔRv2569c+Rv2569c |
| --- | --- | --- |
| 1600 | 1040 | 1067 |
| 1627 | 533 | 1653 |
| 1333 | 347 | 1547 |
| 1920 | 747 | 1307 |

Fig 6D Bacterial loads of the spleen in the mice were quantified by colony-counting at 30 days and 45 days after infection

30 d (CFUs)

| H37Rv | H37RvΔRv2569c | H37RvΔRv2569c+Rv2569c |
| --- | --- | --- |
| 6320 | 560 | 880 |
| 11200 | 520 | 600 |
| 12120 | 240 | 400 |
| 7440 | 280 | 320 |

45 d (CFUs)

| H37Rv | H37RvΔRv2569c | H37RvΔRv2569c+Rv2569c |
| --- | --- | --- |
| 7680 | 3000 | 4760 |
| 8680 | 1320 | 4520 |
| 8400 | 560 | 3200 |
| 9960 | 1040 | 3800 |

Fig 7B Gross pathology score of the lung.

| H37Rv | H37RvΔRv2569c | H37RvΔRv2569c+Rv2569c |
| --- | --- | --- |
| 4 | 2 | 3 |
| 4 | 1 | 2 |
| 4 | 1 | 2.5 |
| 3 | 1 | 2 |

Fig 7D Lung histopathology score

| H37Rv | H37RvΔRv2569c | H37RvΔRv2569c+Rv2569c |
| --- | --- | --- |
| 4 | 2 | 3 |
| 3 | 1.5 | 3 |
| 3 | 1.0 | 2.5 |
| 3 | 2 | 2 |

Fig 8A Western blot analysis of the expression of E-cadherin in the lung after challenge with H37Rv, H37RvΔRv2569c, and H37RvΔRv2569c + Rv2569c at 14 days, 30 days, and 45 days after infection.


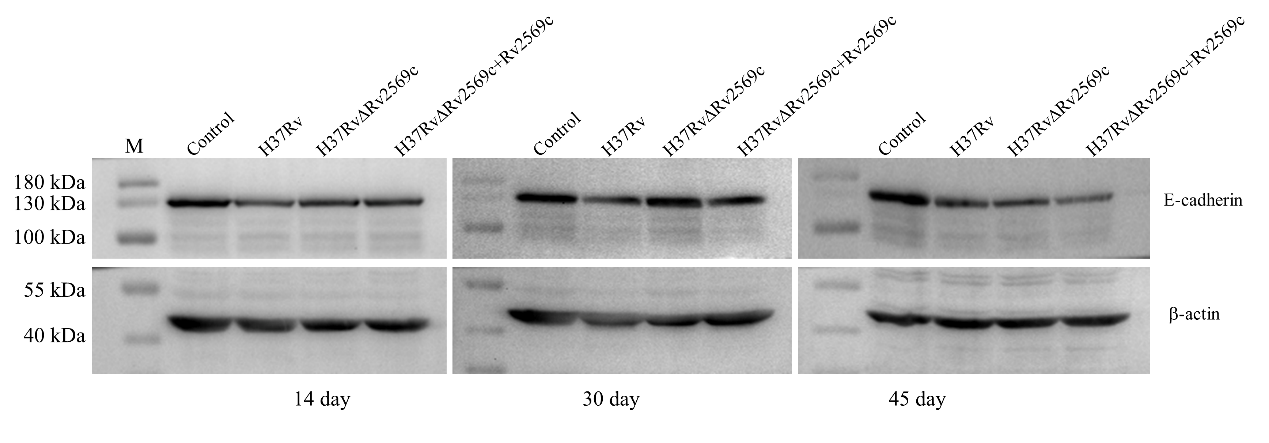


Fig 8B Relative protein expression of E-cadherin in the lung based on β-actin measured by ImageJ

14 d (gray value: E-cadherin/β-actin)

| Control | H37Rv | H37RvΔRv2569c | H37RvΔRv2569c+Rv2569c |
| --- | --- | --- | --- |
| 0.8754 | 0.6835 | 0.7954 | 0.7506 |
| 0.8117 | 0.6461 | 0.7389 | 0.6907 |
| 0.8743 | 0.6883 | 0.7932 | 0.7557 |

30 d

| Control | H37Rv | H37RvΔRv2569c | H37RvΔRv2569c+Rv2569c |
| --- | --- | --- | --- |
| 0.9185 | 0.7814 | 0.9181 | 0.7847 |
| 0.8218 | 0.7146 | 0.8616 | 0.7547 |
| 0.9178 | 0.8150 | 0.9510 | 0.8200 |

45 d

| Control | H37Rv | H37RvΔRv2569c | H37RvΔRv2569c+Rv2569c |
| --- | --- | --- | --- |
| 0.9195 | 0.5997 | 0.5934 | 0.5454 |
| 0.9220 | 0.5990 | 0.5735 | 0.4782 |
| 0.9565 | 0.6525 | 0.6049 | 0.4613 |
